# A high-throughput screening identifies MCM chromatin loading inhibitors targeting cells with increased replication origins

**DOI:** 10.1016/j.isci.2024.110567

**Published:** 2024-07-22

**Authors:** Lucia Falbo, Hervé Técher, Vincenzo Sannino, Michela Robusto, Giovanni Fagà, Federica Pezzimenti, Francesco Romeo, Luca Gabriele Colombo, Stefania Vultaggio, Daniele Fancelli, Silvia Monzani, Valentina Cecatiello, Sebastiano Pasqualato, Mario Varasi, Ciro Mercurio, Vincenzo Costanzo

**Affiliations:** 1 IFOM-ETS, The AIRC Institute of Molecular Oncology, Milan, Italy; 2 Department of Oncology and Hematology-Oncology, University of Milan, 20133 Milan, Italy; 3 Department of Experimental Oncology, European Institute of Oncology (IEO) IRCCS, 20141 Milan, Italy

**Keywords:** Biochemistry, Molecular biology, Cell biology

## Abstract

Replication origin assembly is a pivotal step in chromosomal DNA replication. In this process, the ORC complex binds DNA and, together with the CDC6 and CDT1, promotes the loading of the MCM helicase. Chemicals targeting origin assembly might be useful to sensitize highly proliferative cancer cells. However, identifying such compounds is challenging due to the multistage nature of this process. Here, using *Xenopus laevis* egg extract we set up a high-throughput screening to isolate MCM chromatin loading inhibitors, which led to the identification of NSC-95397 as a powerful inhibitor of replication origin assembly that targets CDC6 protein and promotes its degradation. Using systems developed to test selective drug-induced lethality we show that NSC-95397 triggers cell death both in human cells and *Xenopus* embryos that have higher proliferative ability. These findings demonstrate the effectiveness of molecules disrupting DNA replication processes in targeting hyperproliferating cells, highlighting their potential as anti-cancer molecules.

## Highlights

• *Xl* egg extract is a powerful tool to identify replication origin assembly inhibitors
• MCMs chromatin loading is a useful readout to screen for DNA replication inhibitors
• NSC-95397 induces CDC6 protein degradation preventing MCM chromatin loading
• NSC-95397 promotes cell death in highly proliferative human cells and *Xenopus* embryos

## Graphical abstract

**Figure:**
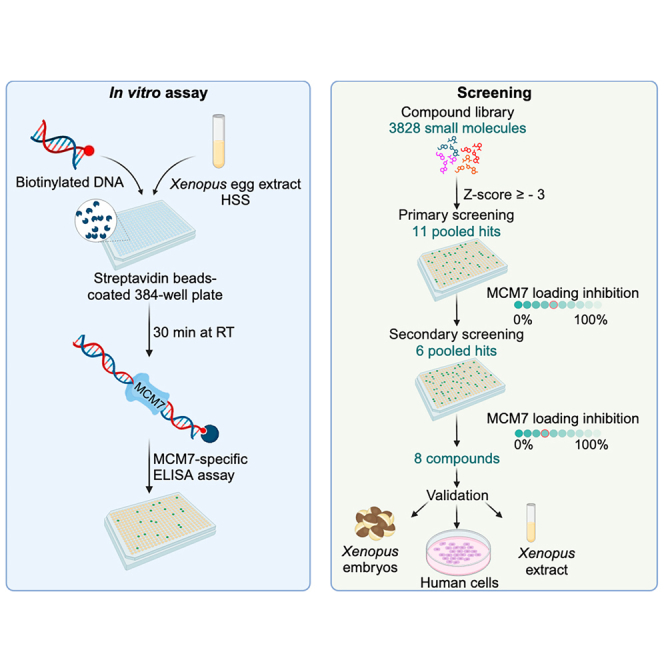

## Introduction

In eukaryotic cells, DNA replication starts at multiple origins distributed throughout the genome. These origins serve as assembly sites for all multiprotein complexes involved in the cell cycle, promoting the formation of bidirectional replication forks. During this phase, the six-subunit origin recognition complex (ORC; subunit ORC1-6) binds DNA and, together with the cell division cycle 6 (CDC6) and the cell division cycle 10-dependent transcript 1 (CDT1), promotes the loading of minichromosome maintenance 2–7 (MCM2-7) double hexamers, which encircle the double-stranded DNA.^1^ This reaction, also known as licensing, takes place at the end of mitosis and during the G1 phase when the inhibitor of replication origin assembly, Geminin, is degraded and the activities of CDK1 and CDK2 are low.^2^ Once in the S-phase, CDK2 activity promotes the binding of CDC45 and the GINS complex to the MCM2-7 double hexamers to form the replicative CMG (Cdc45-MCM-GINS) the activation of which also requires the activity of DBF-dependent kinase (DDK).^3^

Intriguingly, the number of DNA replication origins assembled onto DNA is higher than the ones used in each cell cycle, with the majority remaining dormant. The excess of DNA replication origins allows cells to efficiently and accurately duplicate DNA even when DNA damage irreversibly halts fork progression.^4^ This is particularly useful to bypass stalling events blocking the duplication of a defined DNA segment by two stalled converging forks. In this case activation of dormant origins between the convergent forks can promote duplication of the affected segment by promoting the formation of additional forks moving outwards toward the stalled ones.^5^

Alterations in pathways controlling replication licensing correlate with active cell proliferation, as evidenced by elevated levels of CDC6, CDT1, and MCM proteins observed in many cancer types.^6^ Therefore, the identification of a compound capable of interfering with the loading of MCM2-7 onto DNA represents a promising therapeutic strategy that specifically targets the dysregulated DNA replication process in cancer cells.

The assembly of the origin of replication has been well characterized in *Xenopus* egg cell-free extract, a widely used model system to study DNA replication. Following the addition of DNA templates to egg extract, ORC1-6, and MCM2-7 rapidly load onto DNA in a CDC6-dependent fashion and the CMG helicase is formed once the nuclei are assembled.^7^ The *Xenopus* egg cell-free system represents a unique tool to identify replication origin assembly inhibitors through robust biochemical assays.^8^^,^^9^^,^^10^^,^^11^^,^^12^^,^^13^ Using this system, we recently performed a large chemical screening that led to the identification of 2,3-bis(2-hydroxyethylsulfanyl) naphthalene-1,4-dione, also known as NSC-95397, as a powerful inhibitor of DNA replication. NSC-95397 was previously described as an inhibitor of cell proliferation.^14^ Here, we report that NSC-95397 prevents the assembly of MCM2-7 onto chromatin by directly binding CDC6 and promoting its destabilization, acting as a molecular glue. Importantly, NSC-95397 triggered cell death in human cells and *Xenopus* embryos tailored to hyper-proliferate upon SSRP1 overexpression and subsequent increase of replication origin assembly,^8^^,^^9^ demonstrating its effectiveness *in vivo*.

## Results

### An *in vitro* assay to isolate inhibitors of MCM2-7 loading onto DNA

**Figure 1:**
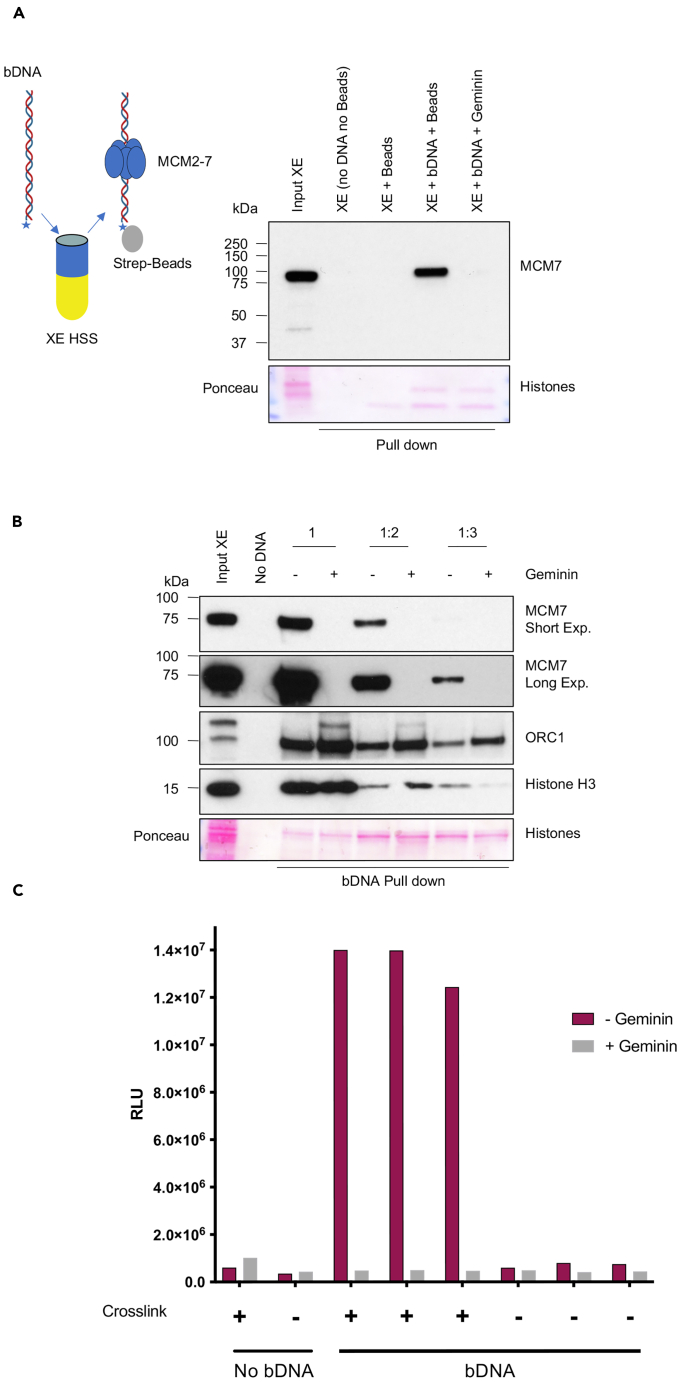
An *in vitro* assay to isolate inhibitors of MCM2-7 loading onto DNA (A) Pull-down. Western blot analysis of interphase *Xenopus laevi*s egg extract incubated for 30 min with or without streptavidin beads bound to a 1.8 kb long DNA molecule biotinylated at the 5′-end (bDNA). MCM2-7 binding to DNA was effectively inhibited by Geminin. No DNA indicates the absence of a DNA template, neither sperm nuclei nor the 1.8 kb long DNA fragment. (B) Pull-down of biotinylated DNA (bDNA) was performed at 30 min. HSS was diluted with XB buffer two-to 3-fold in the presence or absence of the recombinant protein Geminin. No DNA indicates the absence of nuclei. (C) Luminescence w/wo geminin. ELISA assay was performed in a 384-well plate using a 2-fold diluted *Xenopus* extract, in the presence or absence of the recombinant protein Geminin, as well as a crosslinking reagent (formaldehyde). Luminescence was acquired with the Enspire plate reader and it is expressed as Relative Luminescence Unit (RLU). The graph shows a representative experiment.

To identify inhibitors of replication origin assembly, we used high-speed supernatant (HSS) *Xenopus laevi*s egg extracts arrested in interphase, which can rapidly assemble ORC1-6 and MCM2-7 complexes on chromatin following the addition of DNA templates.^15^ Streptavidin beads bound to a 2 kilobases (kb) long DNA molecule biotinylated at the 5′ end were incubated in HSS for 30 min and then recovered. After stringent washing MCM2-7 assembly onto DNA could be detected by western blot of the MCM7 subunit. MCM2-7 binding to DNA was effectively inhibited by Geminin (Figure 1), indicating its physiological assembly.

To optimize this assay for drug screening purposes, we tested the ability of diluted extracts to support the loading of MCM2-7 on DNA. We found that a two-to 3-fold dilution of HSS with egg extract buffer preparation (XB) was still able to support Geminin-sensitive MCM2-7 DNA loading, including all previous reactions leading to chromatin formation and ORC1-6 DNA binding (Figure 1B). This optimization allowed to minimize the egg extract production for the screening of a large number of small molecules.

### Setting up a small molecule screening to isolate inhibitors of MCM2-7 loading onto DNA

Once the MCM2-7 DNA loading conditions were validated by WB, we adapted this *in vitro* binding assay to an enzyme-linked immunosorbent assay (ELISA) suitable for the 384-well plate format. To this end, 1.8 kb long biotinylated DNA (bDNA), working as a scaffold for the ORC-MCM loading, was generated and immobilized on the bottom of a streptavidin-coated 384-well plate. Then, 2-fold diluted HSS was added in each well in the presence or absence of the recombinant protein Geminin. After 30 min of incubation at room temperature, wells were washed twice with a washing buffer, and then the bDNA-protein complexes were cross-linked before being processed with an MCM7-specific ELISA assay. The stable loading of MCM2-7 onto bDNA was detected using a primary antibody specific to MCM7, which, in turn, was recognized by an HRP-labeled secondary antibody. The addition of the chemiluminescence HRP substrate allows a quantitative luminescent readout of antibody binding and, consequently, of the loading of MCM2-7 on bDNA. The ELISA-based assay was extremely sensitive and specific (signal-to-background ratio greater than 10) in revealing the MCM2-7 loading onto bDNA, as well as the ability of Geminin to inhibit it (Figure 1C).

**Figure 2:**
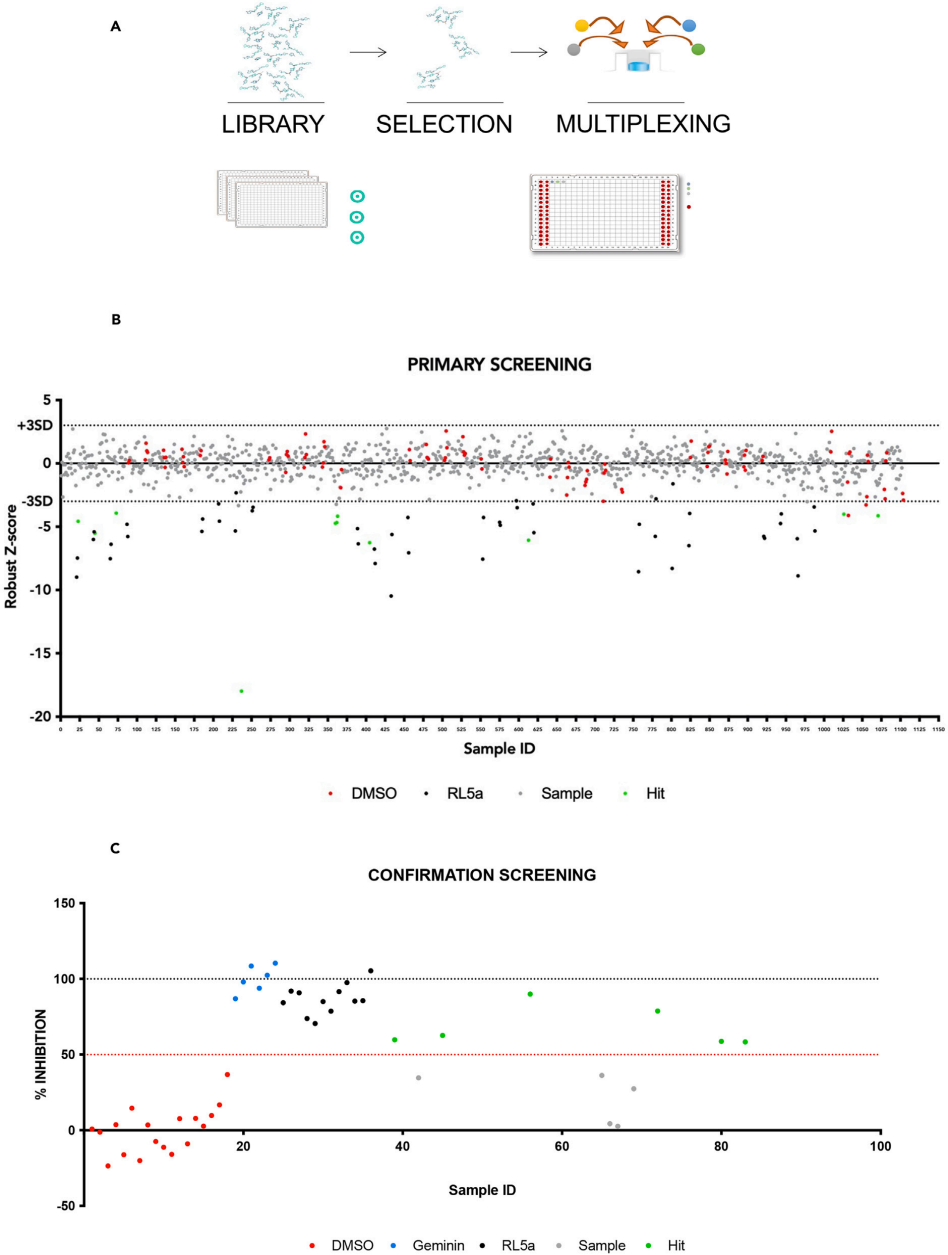
Setting up a small molecule screening to isolate inhibitors of MCM2-7 loading onto DNA (A) HTS workflow outline. (B) Scatterplot of the primary screening data. The plot of Z-scores for each of the pooled compounds subjected to the primary screen for the identification of an inhibitor of MCM2 loading to bDNA. Values represent a single replicate per compound concentration. The screening was performed on 384-well plates where negative controls (DMSO) are reported in red, positive controls are reported in black (RL5a), and hits are in green. Solid lines represent the mean chemiluminescent value for the measurements of the controls and compounds, and dashed lines denote the average ±3 standard deviation of the negative and positive controls, respectively. (C) Hit confirmation. The pooled compounds were screened in triplicate. Geminin and RL5A were used as positive controls, while DMSO corresponds to the negative control. Data are expressed as the mean percentage of inhibition of MCM7 loading on bDNA compared to vehicle (DMSO). The dashed red line corresponds to the threshold of 50% inhibition. See also Figure S1.

Using this assay, were screened 3828 small molecules for their ability to impact the MCM2-7 loading on DNA (Figure 2A). The molecules selected for the screening represented most of the chemical classes of the in-house collection (ca. 215.000 small molecules) available at the Experimental Therapeutic Program of IFOM. The subset selected for this screening included clinically approved drug molecules, compounds belonging to a kinase-targeting library, and molecules designed to hamper protein-protein interaction to embrace an ample variety of structural chemotypes and biological activities among the targets.

To minimize the amount of extract to use, the 3828 compounds were tested as pools of four compounds per well at a fixed concentration of 187 μM each to match the extract’s high protein concentration. In each multiwell plate RL5a, a known licensing inhibitor,^16^ and DMSO were used as positive and negative controls, respectively. In addition, wells without bDNA were used to define the background level of the measured luminescent signal.

The screening was carried out in three rounds and the hits were identified by applying a robust *Z* score approach, which was chosen based on the normal distribution of the curve of the experimental data (Figure S1A). After *Z* score normalization, the majority of the compounds did not inhibit the MCM binding to bDNA and consequently appeared grouped with the DMSO controls (Figure 2B). This “inactive” set was separated from the positive controls and a small number of experimental wells that resulted within the range of the positive controls. Hits were then defined as those molecules (wells) that could reduce the MCM binding to bDNA at least three standard deviations higher than the overall mean of the wells (*Z* score ≥ - 3). This approach led to the identification of 11 pooled hits with a hit identification rate of 1.1%.

The 11 pooled hits were then tested in triplicate at the dose of 187 μM for confirmation. As reported in the graph (Figure 2C), six of them were confirmed to be able to reduce the MCM chromatin loading on bDNA by at least 50% compared to the DMSO. Finally, the 6 hits pools were deconvoluted by testing the corresponding 24 compounds as single molecules in triplicate, at the dose of 187 μM (Figure S1B). The deconvoluted compounds able to reduce MCM chromatin loading on bDNA by at least 40% compared to the DMSO turned out to be Chelerythrine, Candicidin, Reactive blue 2, NSC-95397, DDP-28574, valsartan, aminacrine, and ethacridine (Figure S1C). After an initial analysis of the structures and activity profiles of these hits, the nonspecific cytotoxic compound Chelerythrine was deprioritized, as well as Candicidin, which is a mixture of antifungal heptaene macrolides and Reactive blue 2, for its non-specific affinity for nucleotide-binding regions present in a variety of proteins.

### Validation of the identified compounds

**Figure 3:**
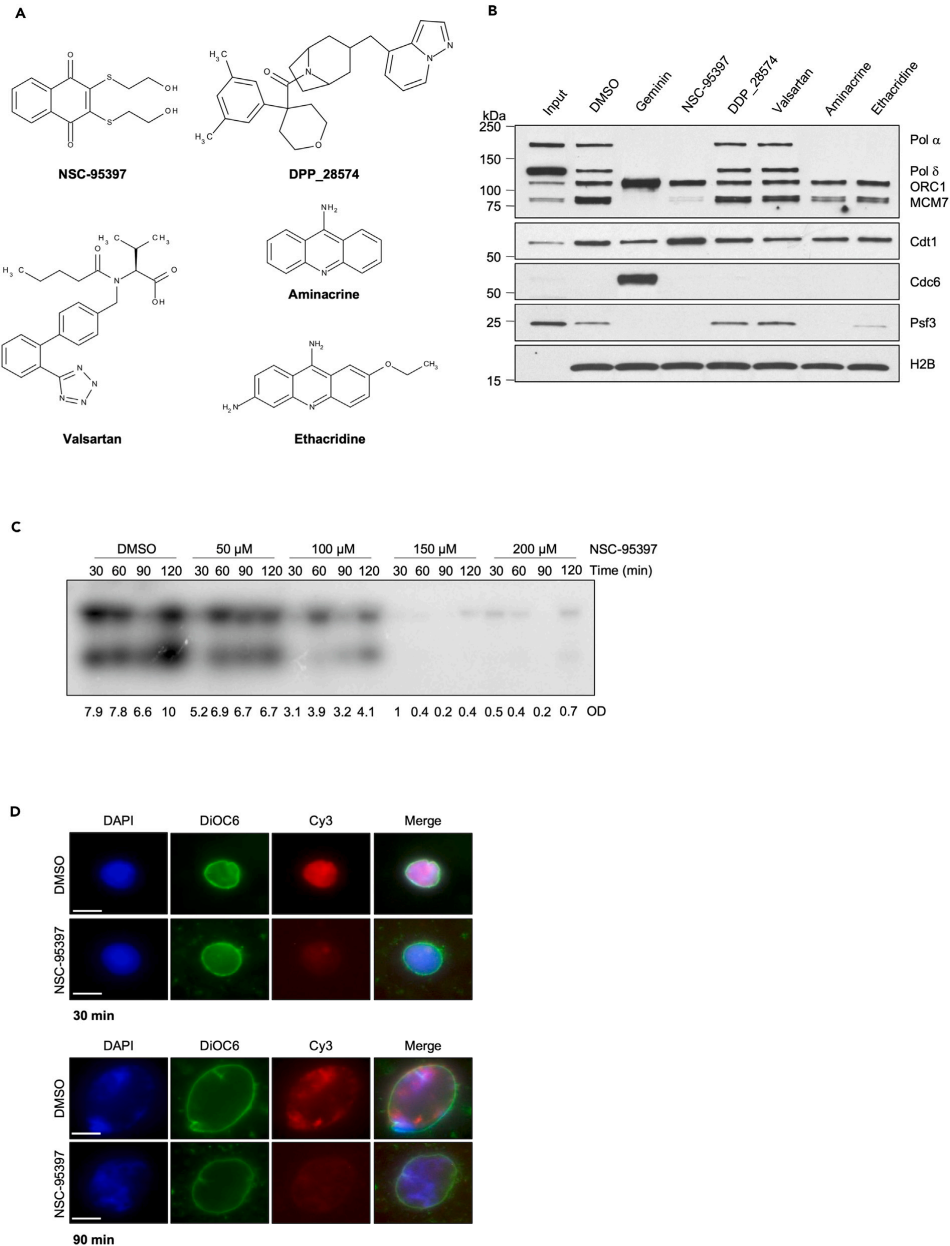
Validation of the identified compounds (A) Chemical structure of the 5 hits identified in the compound screening: NSC-95397, DDP_28574, valsartan, aminacrine, and ethacridine. (B) Western blot analysis of the indicated proteins bound to chromatin isolated from sperm nuclei incubated with DMSO, used as Control, Geminin, or 200 μM NSC-95397, DDP_28574, valsartan, aminacrine, ethacridine for 30 min. (C) Autoradiography of a DNA replication assay showing the time course of α^32^P-dCTP incorporation in sperm nuclei incubated in egg extract treated with DMSO or with increasing concentrations of NSC-95397 (50-100-150-200 μM). Samples were taken at the indicated times after nuclei and α^32^P-dCTP addition to egg extracts. Optical density (OD) for each lane is indicated. (D) Representative images of sperm nuclei incubated in interphase extract supplemented with Cy3-dCTP (red) for 30 min (Upper panel) or 90 min (Lower panel) and treated with DMSO, used as control, or 200 μM NSC-95397. Samples were fixed and stained with 4,6-diamidino-2-phenylindole (DAPI) for DNA (blue) and DiOC6 (green) for membranes. Scale bar = 10 μm. See also Figure S2.

To verify the impact of the remaining five hits, including NSC-95397, DDP-28574, valsartan, aminacrine, and ethacridine (Figure 3A; Table 1) on licensing we used undiluted low-speed supernatant *Xenopus* egg extracts capable of supporting various processes, such as chromatin formation, origin assembly, nuclear membrane assembly, and origin firing. To evaluate the chromatin loading status of replication proteins in the presence of these compounds, we conducted a chromatin binding assay with a fixed concentration of the drugs (Figure 3B). To avoid any possible interference with sperm chromatin decondensation, the drugs were added 5 min after the beginning of the reaction and before licensing. In this assay, we observed that NSC-95397 was able to potently suppress MCM2-7 loading onto chromatin while aminacrine and ethacridine inhibited MCM loading to a minor extent (Figure 3B). Moreover, compounds such as valsartan and DDP_28574 showed limited effectiveness in preventing MCM loading (Figure 3B).

**Table 1:** List of hits identified from the compounds screening

Compound name	IUPAC name
NSC-95397	2,3-bis(2-hydroxyethylsulfanyl) naphthalene-1,4-dione
DDP_28574	[4-(3,5-dimethylphenyl)tetrahydropyran-4-yl]-[3-(pyrazolo[1,5-*a*]pyridin-4-ylmethyl)-8-azabicyclo[3.2.1]octan-8-yl]methanone
Valsartan	(2S)-3-methyl-2-[pentanoyl-[[4-[2-(2H-tetrazol-5-yl)phenyl]phenyl]methyl]amino]butanoic acid
Aminacrine	acridin-9-amine
Ethacridine	7-ethoxyacridine-3,9-diamine

See also Figure 3A.

To exclude that the observed effect on MCM loading was due to the direct binding to bDNA, we conducted a fluorescent intercalator displacement assay with NSC-95397, aminacrine, and ethacridine (Figure S1D). In this assay, SYBR green binding to bDNA was used as a fluorescent indicator, while bDNA was used as DNA template. Doxorubicin, a well-known DNA intercalator,^17^ was used as a positive control. The displacement of the SYBR green determined a strong decrease in the fluorescent signal readout compared to DMSO. Similarly, aminacrine and ethacridine binding to bDNA displaced SYBR green determining a clear decrease of the fluorescent signal at the dose of 100 μM (Figure S1D). We concluded that aminacrine and ethacridine disrupt MCM loading because of their DNA intercalator properties. Instead, NSC-95397 did not cause a major displacement of SYBR. These results indicate that the effects observed with NSC-95397 on MCM loading are not a consequence of its direct binding to bDNA.

In agreement with the inhibition of MCM2-7 chromatin loading, NSC-95397 affected the binding of DNA polymerases α alpha and δ delta, and the Psf3 component of the GINS complex onto DNA. This impairment of replication machinery assembly is likely a consequence of the absence of MCM2-7 assembly (Figure 3B). Importantly, we noticed that NSC-95397 also prevented the binding of CDC6, a critical component of the licensing reaction, while it did not affect ORC1-6 binding to DNA (Figure 3B). These results collectively indicate that NSC-95397 disrupt the chromatin loading of essential replication proteins such as MCM2-7 and CDC6 while sparing the binding of ORC1-6 to DNA.

In line with these results, α^32^P-dCTP incorporation in nascent DNA measured through standard DNA replication time course was reduced by NSC-95397 in a dose-dependent manner (Figure 3C). DNA replication inhibitory concentrations of NSC-95397 were also able to inhibit MCM2-7 chromatin loading (Figure S2A).

We then monitored fluorescently labeled dNTP incorporation in intact nuclei and found that in NSC-95397-treated extracts, the incorporation of Cy3-dCTP into nascent DNA showed a significant reduction compared to the DMSO-treated control extracts (Figures 3D and S2B), further confirming that NSC-95397 treatment inhibits chromosomal DNA replication in a dose-dependent manner.

These results collectively provided the first insights into the mechanism of action of NSC-95397, demonstrating its early interference with CDC6 binding and its inhibitory effect on DNA replication in *Xenopus* egg extracts.

### NSC-95397 targeting of CDC6

**Figure 4:**
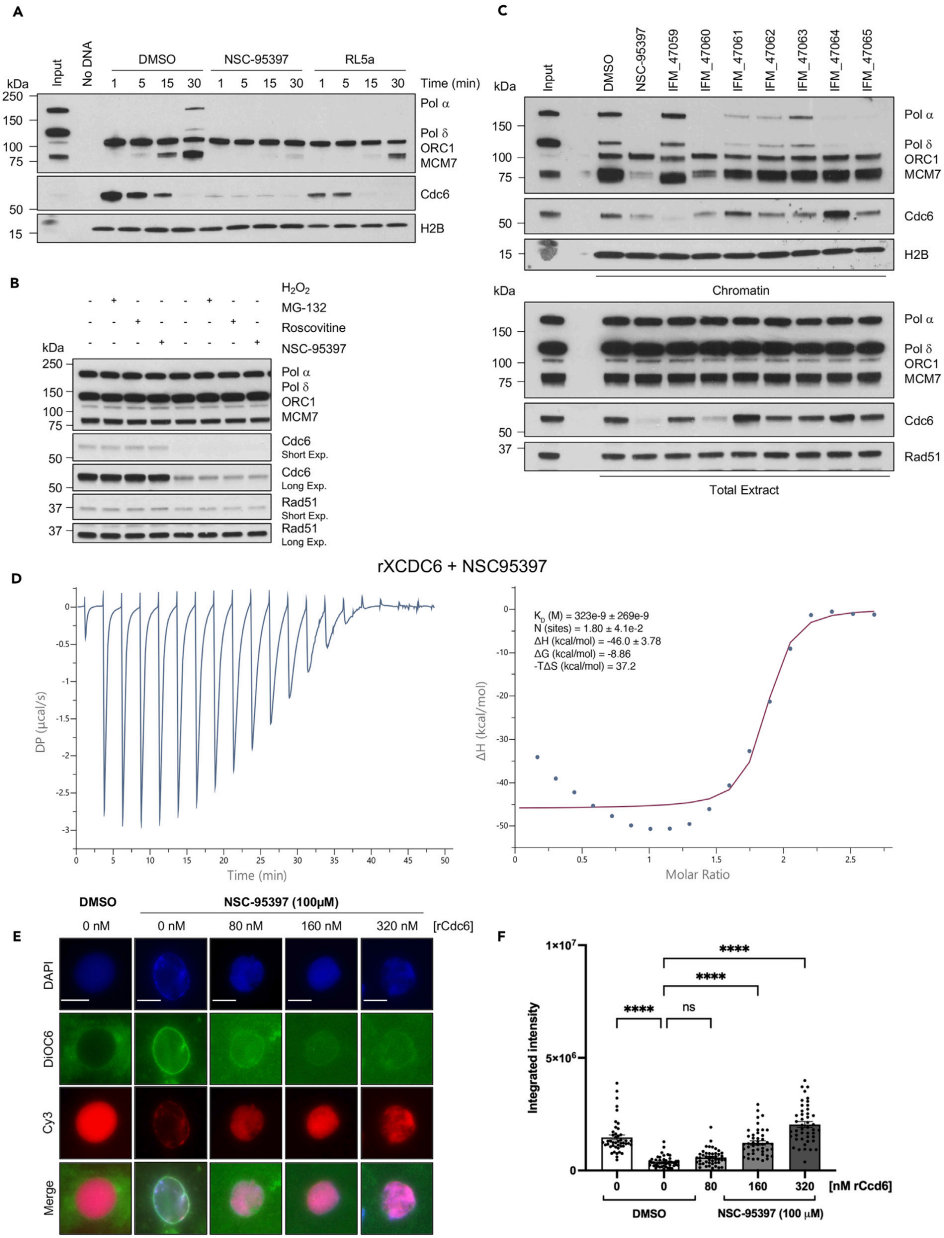
A direct effect of NSC-95397 on CDC6 (A) Western blot analysis of the indicated proteins bound to chromatin isolated at the indicated time (1, 5, 15, 30 min) after sperm nuclei addition in egg extract incubated with DMSO, 200 μM NSC-95397, and 200 μM RL5a. No DNA indicates the absence of nuclei. (B) Western blot analysis of the indicated proteins incubated for 5 min at 23°C with 300 μM H_2_O_2_, 500 μM roscovitine, 200 μM NSC-95397, or 200 μM MG-132 and then kept on ice. (C) Western blot analysis of the indicated proteins bound to chromatin isolated from sperm nuclei incubated for 30 min with DMSO, used as Control, or 200 μM NSC-95397 analogs (IFM_47059, IFM_47060, IFM_47061, IFM_47062, IFM_47063, IFM_47064, IFM_47065). (D) ITC analysis of NSC-95397 binding to recombinant *Xenopus laevis* CDC6. See also Figure S3. (E) Representative images of sperm nuclei incubated in interphase extract supplemented with Cy3-dCTP (red) for 90 min with DMSO, used as control, or 100 μM NSC-95397, in the presence of increasing concentrations of recombinant CDC6 protein (0, 80, 160, 320 nM). Samples were fixed and stained with 4,6-diamidino-2-phenylindole (DAPI) for DNA (blue) and DiOC6 (green) for membranes. Scale bar = 10 μm. See also Figure S4. (F) The Cy3-dCTP incorporation of 45 nuclei was quantified as fluorescence-integrated intensity per nucleus using ImageJ. Data are represented as scatter dot plots. Horizontal bars indicate mean ± SEM. A two-way ANOVA test was used to perform multiple comparisons between DMSO and NSC-treated samples with 0 nM of rCDC6 and between NSC-treated samples with 0 nM of rCDC6 and increasing concentrations of recombinant protein added. ∗∗∗∗ = *p* < 0.0001, ns, not significant.

Having excluded a direct DNA binding effect we performed a time course experiment to elucidate the impact of NSC-95397 on the licensing reaction dynamics and DNA replication (Figure 4A). We found that NSC-95397 prevents CDC6 chromatin loading after only 1 min from the addition of sperm nuclei to egg extract (Figure 4A). We also conducted a comparative analysis between NSC-95397 and the already established RL5a, which is known to prevent origin assembly.^16^ We found that, in contrast to NSC-95397, RL5a suppressed MCM loading without impacting CDC6, in agreement with previous findings^16^ (Figure 4A).

To dissect the molecular mechanism underlying the inhibitory activity of NSC-95397, we initially assessed whether it directly impacted replication protein levels in egg extract under the same conditions employed in our screening. To this end, we incubated interphase egg extract with NSC-95397 for 5 min at room temperature and performed a western blot analysis of the major replication proteins. Surprisingly, we observed a rapid decrease in the total levels of CDC6 in egg extracts treated with NSC-95397. In contrast, the total levels of other DNA binding proteins such as ORC1, MCM7, polymerase α and δ, and the DNA repair protein RAD51 were not affected by the treatment (Figure 4B).

As NSC-95397 is a quinone compound potentially able to induce toxicity by inducing oxidative stress, we tested whether the levels in our experimental settings could be affected by the action of a standard reactive oxygen species (ROS) inducer. To explore this, we exposed the egg extract to 300 μM H_2_O_2_ for 5 min. Notably, this treatment did not affect the CDC6 protein levels, suggesting that the NSC-95397 activity on CDC6 is not mediated by ROS (Figure 4B).

To verify whether CDC6 degradation was dependent on proteasomal-mediated processing, we also inhibited proteasome activity using 200 μM MG-132. As shown in Figure 4B, the treatment of extract with proteasome inhibitor MG-132, either alone or in combination with NSC-95397, failed to increase CDC6 levels. This experiment revealed that NSC-95397 induces CDC6 degradation despite the treatment with proteasome inhibitor MG-132. Thus, the decline in levels of CDC6 induced by NSC-95397 is proteasome-independent and might rely on other proteases degrading unfolded intracellular proteins.^18^

We further tested whether CDC6 degradation upon NSC-95397 is mediated by Cyclin-dependent kinases (CDKs). However, the addition of a CDK inhibitor such as roscovitine did not result in any observable effects on the rate of CDC6 degradation (Figure 4B). This suggests that the degradation of CDC6 triggered by NSC-95397 treatment occurs through a CDK-independent pathway.

To test whether the other quinone compounds were able to destabilize CDC6, we examined the effects of several commercial analogs (Figure S4A; Table 2) on the CDC6 protein levels in interphase egg extract (Figure 4C). We found that only a close structural analog of NSC-95397 IFM_47060 showed some CDC6 protein destabilization (Figure 4C).

**Table 2:** List of the analog compounds of NSC-95397

ID identification	IUPAC name
NSC-95397	2,3-bis(2-hydroxyethylsulfanyl) naphthalene-1,4-dione
IFM_47059	2,3-bis(butylsulfanyl)naphthoquinone
IFM_47060	2,3-bis(methylsulfanyl)naphthoquinone
IFM_47061	3-(3,4-dimethoxyphenyl)-1-ethyl-6-methylpyrimido[5,4-e][1,2,4]triazine-5,7(1H,6H)-dione
IFM_47062	2-((2-methoxyethyl)thio)-3-methylnaphthalene-1,4-dione
IFM_47063	2-methyl-3-(methylsulfanyl)naphthoquinone
IFM_47064	3-(4-fluorophenyi)-1,6-dimethylpyrimido[5,4-e][1,2,4]triazine-5,7(1H,6H)-dione
IFM_47065	2-(2-Hydroxyethylthio)-3-methylnaphthalene-1,4-dione

See also Figures 4C and S4A.

To assess whether CDC6 destabilization was due to the direct interaction of NSC-95397 with CDC6, we expressed *Xenopus laevis* recombinant CDC6 protein (rCDC6) (Figures S3A and S3B) and performed isothermal titration calorimetry (ITC) experiments.^19^ The results of ITC shown in the graph on the left of Figure 4D display the differential power (DP) in microcalories per second plotted against time in minutes, with characteristic exothermic peaks indicating binding events.^19^ The panel on the right instead shows the integrated heat data (ΔH in kilocalories per mole) plotted against the molar ratio of NSC-95397 to CDC6, with the data points fitting a sigmoidal curve typical of a binding isotherm. The experiment was conducted at a fixed CDC6 concentration (74 μM) with titrations of NSC-95397 up to 1 mM. The fit parameters suggest a single set of binding sites with a dissociation constant (K_D_) in the nanomolar range (323 ± 269 nM), indicating a strong interaction, and a negative enthalpy (ΔH) change (−46 ± 3.78 kcal/mol) consistent with an energetically favorable binding process. These results indicate that NSC-95397 directly interacts with CDC6 protein *in vitro* possibly causing its destabilization and degradation *in vivo* through non-proteasome-dependent chaperone-controlled pathways.^18^

To confirm that CDC6 is a major target of NSC-95397, we performed a rescue experiment by adding increasing amounts of the rCDC6 protein to overcome the effect of the NSC-95397 and examined the total DNA replication after 90 min of incubation of sperm nuclei with DMSO or with the drug. We observed an NSC-95397-dependent impairment of fluorescent Cy3-dCTP incorporation in intact nuclei that was significantly prevented by increasing concentrations of rCDC6 (Figures 4E and 4F). Similar results were obtained by monitoring the α^32^P-dCTP incorporation in replicating DNA (Figure S4B). The partial rescue observed in both assays may be possibly attributed to NSC-95397 potentially affecting additional targets involved in DNA replication besides CDC6.

Importantly, consistent with NSC-95397 mediated targeting of CDC6, which acts early in the replication origin assembly reaction, NSC-95397 added to interphase egg extracts 20 min after sperm nuclei, when the replication origins had already formed, was unable to inhibit DNA replication (Figures S4C and S4D). These results confirmed that NSC-95397 inhibits the early step of the assembly of DNA replication origins mediated by CDC6.

### Effects of NSC-95397 on *Xenopus* development

**Figure 5:**
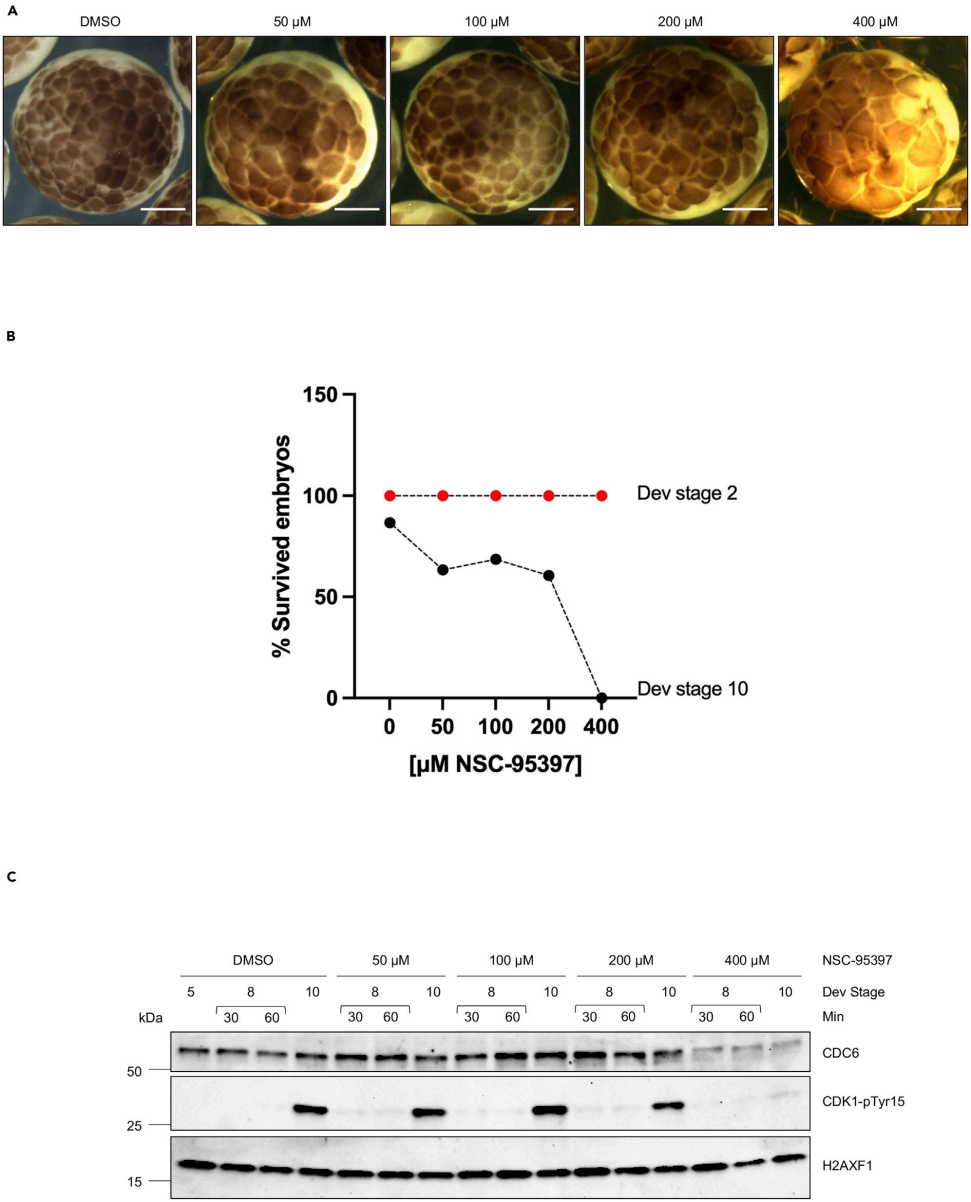
Effect of NSC-95397 during early development (A) *Xenopus* eggs were subjected to *in vitro* fertilization (IVF) and then incubated in the absence (DMSO) or in the presence of NSC-95397 (50 μM, 100 μM, 200 μM or 400 μM), and the survival rate was assessed at 8 h after IVF. Scale bar = 500 μm. (B) The percentage of survived embryos at developmental stage 10 was normalized to the total number of eggs that underwent IVF. (C) Western blot of whole embryos incubated in the absence (DMSO) or in the presence of NSC-95397 (50 μM, 100 μM, 200 μM or 400 μM) and taken at the indicated developmental stages. Stage 8 was sampled every 30 min.

To validate the effects of NSC-95397 on the cell cycle *in vivo* we investigated its effects on developing embryos following *Xenopus laevis* eggs fertilization. Embryos reaching the two-cell stage were arrayed into 6-well plates (100 eggs per well) and exposed to increasing concentrations of NSC-95397 (50–400 μM) and the appropriate control (DMSO) (Figure 5A) placed in a developing buffer. The embryos were left to develop until stage 10, which is reached after few cell divisions.^9^ We then analyzed the survival rate and the expression levels of CDC6 protein of the developing embryos (Figure 5). NSC-95397 did not show apparent toxicity below 200 μM (Figure 5A). Above this concentration, the compound altered the cleavage of the eggs and the survival ratio. The embryos grown in the presence of NSC-95397 showed a significant dose-dependent increase in mortality (Figure 5B). As shown in Figure 5C, WB analysis indicated that NSC-95397 at high doses (400 μM) induced a significant downregulation of CDC6 correlating with a decrease in the Cdk1 Tyr15 phosphorylation (CDK1-pTyr15) normally associated to active S-phase.^20^ These findings underscore the dose-dependent impact of NSC-95397 on both embryonic survival and CDC6 protein levels, suggesting its potential as a regulator of DNA replication and cell cycle progression processes during *Xenopus laevis* development. NSC-95397 has been reported to inhibit Cdk1 Tyr-15 phosphatase CDC25.^14^ Although we cannot completely exclude an effect on CDC25, we did not observe increased Cdk1 Tyr-15 phosphorylation induced at the midblastula transition^9^ as would be expected by the inhibition of CDC25, which normally removes Tyr-15 phosphorylation. These results suggest that the action of NSC-95397 on developing *Xenopus* embryos is mostly independent of its known effect on CDC25 (Figure 5C).

### Effects of NSC-95397 on cells and embryos with increased DNA replication origin assembly

**Figure 6:**
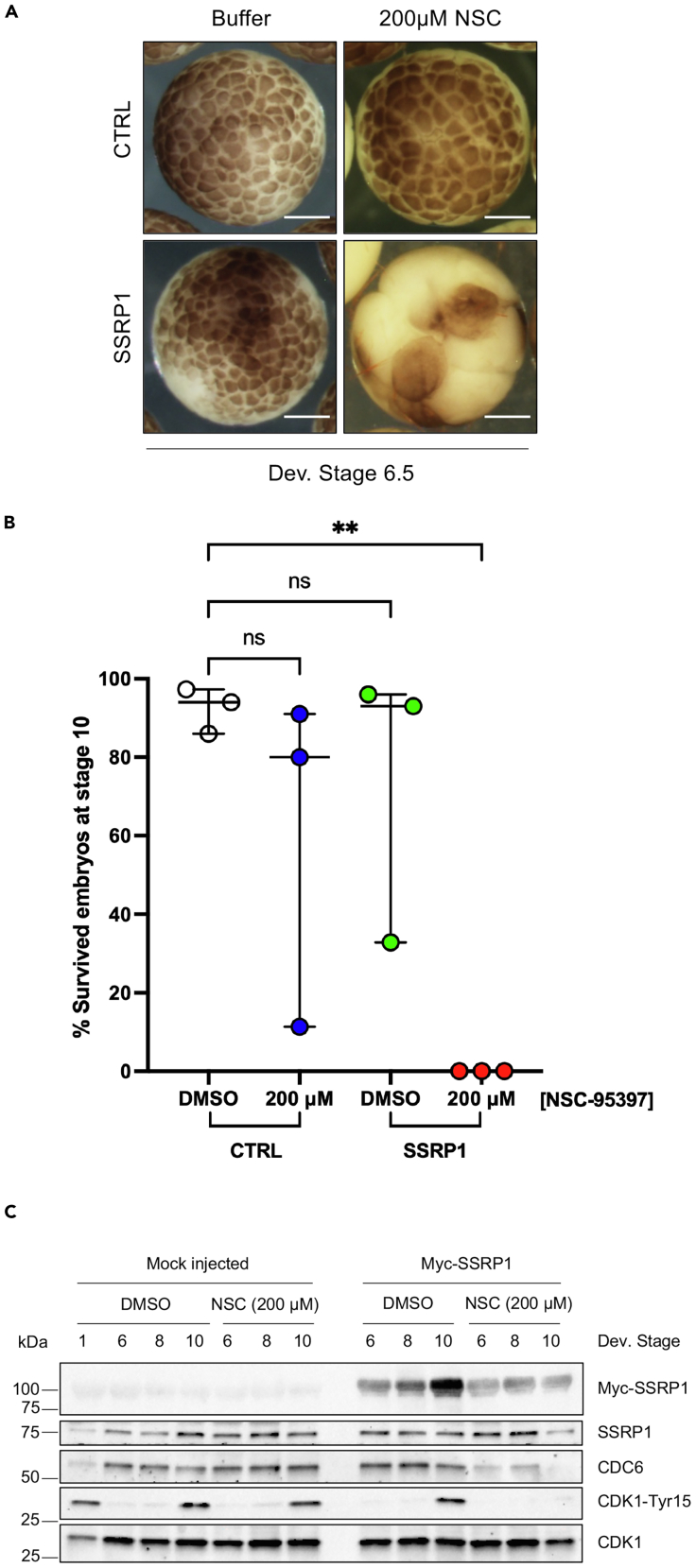
Effects of NSC-95397 in SSRP1 overexpression conditions (A) Images of embryos injected with water (control, first row) or SSRP1 mRNA (second row) and then incubated in the absence (DMSO) or in the presence of 200 μM NSC-95397. Scale bar = 500 μm. See also Figure S5. (B) The percentage of survived embryos at developmental stage 10 was normalized to the total number of eggs that underwent IVF. The results from independent experiments are expressed as the raw percentage of embryos at each developmental stage. Horizontal bars indicate the median with range. One-way ANOVA test was used to analyze differences among multiple groups compared to the control (DMSO). ∗∗ = *p* < 0.0097, ns = not significant. (C) Western blot of whole embryos injected with water, or Myc-SSRP1 mRNA, treated without (DMSO) or with 200 μM NSC-95397 and taken at the indicated developmental stages.

To be useful a drug interfering with DNA replication should be able to preferentially target cancer cells, which tend to have higher levels of replication origins assembled onto chromatin.^21^ We hypothesized that higher levels of replication origins confer intrinsic vulnerability to drugs interfering with DNA replication. To reproduce this condition in a controlled fashion we set up assays based on *Xenopus laevis* embryos and human cells overexpressing the chromatin factor SSRP1. SSRP1 is highly expressed in cancer and potently enhances replication origin assembly as we have previously shown.^9^ Using these systems we tested whether low doses of NSC-95397 confer an intrinsic vulnerability to cells addicted to higher levels of replication origin assembly. Consistent with this hypothesis, we observed that the low doses of NSC-95397 tolerated in control embryos were severely cytotoxic in SSRP1-injected embryos, resulting in early embryonic lethality within 8 h of treatment (Figures 6A and 6B). The phenotypes observed were highly reproducible as all SSRP1-injected embryos treated with the NSC-95397 showed the same phenotype involving early embryonic death.

WB analysis indicated that low doses of NSC-95397, which were tolerated by normal embryos (Figures 5A–5C), induced a significant downregulation of CDC6 protein levels correlating with a decrease in the Cdk1 Tyr-15 phosphorylation normally associated with the onset of midblastula S-phase in SSRP1 injected embryos (Figure 6C). These findings confirmed selective cytotoxic effects of NSC-95397 in cells with high levels of replication origins.

We next asked whether the *in vivo* activities of the compound NSC-95397 extended also to human cells. To investigate this aspect, we used immortalized, non-tumorigenic MCF10A and hTERT-RPE1 cells to construct SSRP1 inducible expression model systems (Figures S5A and S5B). We conducted cell proliferation assays on MCF10A-SSRP1 and hTERT-RPE1-SSRP1 human cells following exposure to different concentrations of NSC-95397 for 48 h (Figures S5C and S5D). Consistent with the embryo results, we observed a significant level of cytotoxicity induced by NSC-95397 (Figures S5C and S5D), enhanced in cells overexpressing SSRP1 as shown by the evaluation of the relative IC50 (Figures S5E and S5F). Similar findings were obtained in HCT116, SW620, and MCF7 cancer cells selected among lines naturally expressing high levels of SSRP1 (Figures S5G and S5H). These results suggest that NSC-95397 induces increased lethality in cells with increased levels of SSRP1 and, possibly, high levels of DNA replication origins assembled onto chromatin. These observations highlight the importance of targeting DNA replication origins assembly processes as an anti-proliferative strategy.

## Discussion

DNA replication is tightly controlled by a process known as origin licensing which ensures the proper duplication of the genome during cell cycle progression. Aberrant expression of proteins involved in the licensing process is associated with replication stress, leading to under- or re-replicated DNA, thus resulting in the occurrence of genomic instability typical of cancer cells.^22^^,^^23^^,^^24^^,^^25^^,^^26^ Indeed, some studies have shown that neoplastic tumor growths often exhibit an increased expression of replication licensing factor CDC6, responsible for the loading of MCM proteins onto replication origins.^27^^,^^28^^,^^29^^,^^30^ In addition, CDC6 overexpression has been shown to promote aggressiveness in cervical,^28^ bladder,^27^ and brain tumors.^31^

Deregulated levels of MCM2-7 complex subunits are also closely associated with cancer.^32^^,^^33^^,^^34^ In cancer cells, the excess of MCMs on chromatin serves as a crucial survival mechanism by activating dormant origins as backups during DNA replication, particularly in the presence of replicative stress.^35^^,^^36^ Conversely, a reduction or deficiency in MCMs to a limited extent, does not impair DNA replication.^24^^,^^37^^,^^38^ However, in the presence of replicative stress lack of sufficient amount of replication origins assembled onto chromatin challenges the completion of DNA replication, giving rise to genomic instability.^35^^,^^36^ Therefore, cancer cells become addicted to high levels of MCMs and other licensing factors. Such addiction generates a vulnerability that could be selectively exploited to kill cells with high levels of replication origins typically associated with high levels of MCM and CDC6 protein expression.

Here, we identified small molecules with the ability to interfere with the binding of the MCM2-7 complex to DNA, a crucial step in DNA replication origin assembly. In particular, we discovered that a compound, known as NSC-95397, a quinone-based small molecule, directly targets CDC6 protein inducing its destabilization, and preventing MCM complex binding to DNA.

NSC-95397-mediated decreased levels of CDC6 in egg extracts are independent of proteasomal degradation and are not affected by the activity of CDKs. This suggests a unique mechanism of CDC6 degradation induced by NSC-95397.

NSC-95397 was first shown to have anti-proliferative potential in several cancers^39^^,^^40^ by promoting inhibition of CDC25 dual-specificity phosphatases.^14^ Here we show that NSC-95397 also inhibits replication origin assembly, triggering cytotoxicity without increasing the levels of CDK1 phosphorylation as expected by an inhibitory effect on CDC25 activity. Any possible relation between CDC6 destabilization and CDC25 activity regulation by NSC-95397 requires further work to be better understood.

To validate the effects of NSC-95397 *in vivo* we set up an assay based on the overexpression of SSRP1, a subunit of the FACT complex, which we have previously shown to remove histone H1 from chromatin promoting increased ORC- and MCM-dependent replication origin assembly.^9^ SSRP1 is often overexpressed in several tumors and this correlates with increased replication origin activity. Accordingly, SSRP1 accelerates DNA replication, cell cycle, and embryo growth, rendering the cells addicted to elevated levels of MCMs assembled onto the chromatin.^9^^,^^41^ Consistent with this, NSC-95397 specifically killed embryos expressing high levels of SSRP1 possibly by targeting cells addicted to high levels of the MCMs loaded on chromatin. This lethality suggests the potential of NSC-95397 in SSRP1 overexpressing tumors and in general in cells that have increased replication origins.

In agreement with the embryo results, we found reduced viability of immortalized and transformed cancer cells overexpressing SSRP1 after the treatment with NSC-95397. Collectively, these results suggest that NSC-95397-mediated targeting of replication origin assembly through the destabilization of CDC6 could be exploited as a powerful strategy to selectively kill highly proliferative cancer cells. The discovery that inhibitors of DNA replication origins assembly induce growth arrest and eventually death of hyperproliferating cells offers promising opportunities for targeted therapies.

### Limitations of the study

The mechanism that leads to proteasome-independent Cdc6 degradation induced by NSC-95397 remains to be identified.

## STAR★Methods

### Key resources table

**Table:** 

REAGENT or RESOURCE	SOURCE	IDENTIFIER
**Antibodies**		

Anti-CDC45 (Rabbit polyclonal)	Aze et al.^42^	N/A
Anti-ORC1 (Mouse monoclonal)	Aze et al.^42^	N/A
Anti-CDK1 (Mouse monoclonal) [A17]	Falbo et al.^9^	N/A
Anti-phospho-Cdk1 (Thr14, Tyr15) (Mouse monoclonal) [CP3.2]	Falbo et al.^9^	N/A
Anti-MCM7 (Mouse monoclonal)	Santa Cruz Biotechnology	Cat#sc-9966; RRID: AB_627235
Anti-CDT1 (Mouse monoclonal)	Santa Cruz Biotechnology	Cat#sc-365305; RRID: AB_10847805
Anti-PSF3 (Rabbit polyclonal)	Hashimoto et al.^43^	N/A
Anti-SSRP1 (Mouse monoclonal)	Abcam	Cat#Ab26212; RRID: AB_449020
Anti-CDC6 (Rabbit polyclonal)	Santa Cruz Biotechnology	Cat#sc-8341; RRID: AB_638342
Anti-*Xenopus* POL α p180 (Mouse monoclonal) peptide antigen used: VKRLPAVTKPGH	Kolinjivadi et al.^44^	Abmart: clone 13026-1-3/C199
Anti-*Xenopus* POL δ 125 kDa (Mouse monoclonal) peptide antigen used: SSQTKKLRGDWDDD	Kolinjivadi et al.^44^	Abmart: clone 19570-1-1/C316
Anti-Histone H2B (Rabbit polyclonal)	Millipore	Cat#07–371; RRID: AB_310561
Anti-H3 (Rabbit polyclonal)	Abcam	Cat#Ab1791; RRID: AB_302613
Anti-RAD51 (Mouse monoclonal) [14B4]	Abcam	Cat#ab213; RRID: AB_302856
HRP anti-Myc tag antibody [9E10]	Abcam	Cat#ab62928; RRID: AB_955371

**Biological samples**		

*Xenopus* egg extract	This manuscript	N/A

**Chemicals, peptides, and recombinant proteins**		

Bovine Serum Albumin	Sigma-Aldrich	Cat#A7030; CAS 9048-46-8
Calcium ionophore	Sigma-Aldrich	Cat#A23187; CAS 52665-69-7
Chorionic gonadotropin	Sigma-Aldrich	Cat#CG10; CAS 9002-61-3
Creatine phosphate	Roche	Cat#10621714001; CAS 71519-72-7
Creatine phosphokinase	Sigma-Aldrich	Cat#C3755; CAS 9001-15-4
Cy3-dCTP	Amersham Biosciences	Cat#PA53021
Cycloheximide	Calbiochem	Cat#239763; CAS66-81-9
Cytochalasin B	Sigma-Aldrich	Cat#C6762; CAS 14930-96-2
Ficoll® PM-400	Sigma-Aldrich	Cat#F4375; CAS 26873-85-8
Geminin	Aze et al.^42^	N/A
Glutathione Sepharose™ 4 Fast Flow	Cytiva	Cat#17-5132-01
L-Cysteine	Sigma-Aldrich	Cat#30089; CAS 52-90-4
Leupeptin	Sigma-Aldrich	Cat#L2023; CAS 147385-61-3
Lysolecithin	Sigma-Aldrich	Cat#L4129; CAS 9008-30-4
NSC-95397	Calbiochem	Cat#217694; CAS 93718-83-3
2,3-Bis(n-butylthio)-1,4-naphthalenedione	Matrix Scientific	Cat#016562; CAS 671189-54-1
2,3-Bis(methylthio)-1,4-naphthalenedione	Matrix Scientific	Cat#016561; CAS 55699-85-9
3-(3,4-dimethoxyphenyl)-1-ethyl-6-methylpyrimido[5,4-e][1,2,4]triazine-5,7(1H,6H)-dione	Vitas-M Lab	Cat#STK249723
3-(4-fluorophenyl)-1,6-dimethylpyrimido[5,4-e][1,2,4]triazine-5,7(1H,6H)-dione	Vitas-M Lab	Cat#STK235850
2-[(2-Methoxy)ethylthio]-3-methyl-1,4-naphthoquinone	Sigma-Aldrich	Cat# SML0367; CAS 255906-59-3
2-(2-Hydroxyethylthio)-3-methylnaphthalene-1,4-dione	Advanced ChemBlocks Inc.	Cat# O32425; CAS 59147-84-1
Protease inhibitor Set III	Calbiochem	Cat#539134
RL5a	Sigma-Aldrich	Cat#SML2187; CAS 133671-66-6
Spermidine	Sigma-Aldrich	Cat#S0266; CAS 124-20-9
Spermine	Sigma-Aldrich	Cat#S4264; CAS 71-44-3
Sucrose	Sigma-Aldrich	Cat#S0389; CAS 57-50-1
Resource S 6 mL column	Cytiva	Cat#17-1180-01
Superdex® 200 10/300 GL	Cytiva	Cat#17-5175-01
MG-132	Calbiochem	Cat#474790; CAS 133407-82-6
Roscovitine	Calbiochem	Cat#557360; CAS 186692-46-6

**Critical commercial assays**		

Amicon® Ultra Centrifugal Filter, 50 kDa MWCO	Millipore	Cat#UFC8050
Bio-Rad Protein Assay	BioRad	Cat#5000006
Gateway Gene Cloning System	ThermoFisher Scientific	Cat#12535-029
Lipofectamine™ 2000 Transfection Reagent	ThermoFisher Scientific	Cat#11668019
mMessage mMachine kit	ThermoFisher Scientific	Cat#AM1340
Nunc™ 384-well polypropylene plates	ThermoFisher Scientific	Cat#4312
Pierce™ 384-well Streptavidin-coated plates	ThermoFisher Scientific	Cat#15505
SuperSignal ELISA Pico	ThermoFisher Scientific	Cat#37069
WesternBright ECL	Advansta	Cat#K-12045-D50
Wizard® SV Gel and PCR Clean-Up System	Promega	Cat#A9281

**Deposited data**		

Original image, quantifications and datasets	Mendeley and OSF	Mendeley Data: https://doi.org/10.17632/v75czchpjm.1 OSF: https://osf.io/undvk/?view_only=804a7de818c845a79e2fa6f15295c57b

**Experimental models: cell lines**		

HEK293T	ICLC	Cat#HTL04001; RRID: CVCL_0063
hTERT-RPE1	ATCC	Cat#CRL-4000™; RRID: CVCL_4388
hTERT-RPE1 6xMyc_SSRP1	This manuscript	N/A
hTERT-RPE1 Empty vector	This manuscript	N/A
MCF10A	ATCC	Cat#CRL-10317™; RRID: CVCL_0598
MCF10A 6xMyc_SSRP1	This manuscript	N/A
MCF10A Empty vector	This manuscript	N/A

**Experimental models: organisms/strains**		

Rosetta™ (DE3) Competent Cells	Novagen	Cat#70954
*Xenopus laevis* females	Nasco	Cat#LM00535MX
*Xenopus laevis* males	Nasco	Cat#LM00715MX

**Oligonucleotides**		

attb1_6M_for: 5′-GGGGACAAGTTTGTACAAAAAAGCAGGCTACGCG TGCAGGATCCCATCGATTTAAAGCTACCATG-3′	This manuscript	N/A
attb1_6M_rev: 5′-GGGGACCACTTTGTACAAGAAAGCTGGGTACCG GTCCTCCCACACCTCCCCCTG-3′	This manuscript	N/A
pBS_NEST_For: 5′-GTGCACGAGTGGGTTACATC-3′	This manuscript	N/A
pBS_NEST_Rev: 5′-CTCTTCGCTATTACGCCAGC-3′	This manuscript	N/A
pBS_b_For1: 5’-[BtnTg]CACAACATACGAGCCGGAAG-3	This manuscript	N/A
pBS_Rev1: 5′-GTGCACGAGTGGGTTACATC-3′	This manuscript	N/A

**Recombinant DNA**		

Glutathion-S-Transferase (GST)-tagged Xenopus Cdc6 plasmid	Furstenthal et al.^43^	N/A
pBlueScript KS(−)	Agilent	Cat#212208
pGEX-6P-1	Cytiva	Cat#28-9546-48
pDONR™221	ThermoFisher Scientific	Cat#12536-017
pCW57.1	Addgene	Addgene #41393; RRID: Addgene_41393
pCW57.1-6xMYC_SSRP1	This manuscript	N/A
pCS2-6xMYC_SSRP1	Falbo et al.^9^	N/A

**Software and algorithms**		

HiTSeekR online platform	List et al.^45^	N/A
ImageJ	ImageJ	RRID: SCR_003070
ImageQuant™ TL 10.2 analysis software	Cytiva	RRID: SCR_018374
NIS-Elements software	Nikon	https://www.nikoninstruments.com/Products/Software; RRID: SCR_014329
Prism (version 9)	GraphPad	https://www.graphpad.com; RRID: SCR_002798

**Other**		

Bio-Rad ChemiDoc™ XRS+ Imaging System	Bio-Rad	RRID: SCR_014210
Bioruptor®	Diagenode	RRID: SCR_023470
EnSpire® plate reader	PelkinElmer	Cat#2104-0020
Hamilton Microlab STAR Liquid Handling robot	Hamilton	N/A
Typhoon biomolecular imager	Cytiva	Cat#29187193
PEAQ-ITC MicroCalorimeter	Malvern Panalytical	RRID: SCR_023795
Microinjector PicoSpritzer III	Parker	Cat#052-0500-900
Micropipette Puller P-97	Sutter Instrument	Cat#P-97; RRID: SCR_016842
Nikon Ds-Fi1-U2 color camera	Nikon	N/A
Nikon SMZ1500 Stereomicroscope	Nikon	N/A
Olympus BX61 Upright fluorescence microscope	Olympus	SM100211-MG1XY
TLA100.3 fixed-angle rotor	Beckman Optima	Cat#349490
VICTOR3™ V 1420 Multilabel plate reader	Perkin Elmer Wallac	Cat#1420-051

### Resource availability

#### Lead contact

Further information and requests for resources and reagents should be directed and will be fulfilled by the lead contact, Prof. Vincenzo Costanzo (vincenzo.costanzo@ifom.eu).

#### Materials availability

Materials generated in this study will be made available upon request by the lead contact.

#### Data and code availability

• Original unprocessed gels, western blot images, microscopy images, and datasets have been deposited on Mendeley Data and Open Science Framework and are publicly available as of the date of publication. Accession numbers are listed in the key resources table.
• The study does not report any original code.
• Any additional information related to the data reported in this paper is available from the lead contact upon request.

### Experimental model and study participant details

#### Experimental model: *Xenopus laevis*

Eggs derived from *Xenopus laevis* frogs were used as an experimental model system. The collection of eggs from the female frogs was performed in a non-invasive way following chorionic gonadotropin (Sigma-Aldrich, Cat. No. CG10) injections. Occasional surgical procedures were performed on the male frogs to harvest sperm nuclei. Experimental protocols were approved by the IFOM Animal Welfare Committee and the Italian Ministry of Health. The number of animals used was kept to a minimum and was calculated taking into account the number of eggs required to obtain the cytoplasmic extract needed for the experiments described. The animals were kept in highly regulated and monitored conditions with room and water temperatures at 19°C. Basic husbandry requirements were provided by the IFOM *Xenopus* facility.

#### Experimental model: Cell lines

Human retinal epithelial hTERT-RPE1 cells were obtained from ATCC. hTERT-RPE1 cells were grown in Dulbecco’s Modified Eagle Medium/F12 (1:1) (DMEM/F12, Gibco) supplemented with 10% Tet-system approved FBS and 1% penicillin/streptomycin and maintained in 5% CO_2_ at 37°C. Human MCF10A cells were grown in Dulbecco’s Modified Eagle Medium/F12 (1:1) (DMEM/F12, Gibco) supplemented with 5% Horse serum, 10 μg/mL human insulin, 20 ng/mL human EGF, 500 ng/mL Hydrocortisone, 100 ng/mL cholera toxin, 1% penicillin/streptomycin and maintained in 5% CO_2_ at 37°C. Human MCF7, HCT116, and SW-620 cells were obtained from NCI. MCF7, HCT116, and SW620 cells were grown in RPMI 1640, 10% FBS, 5% L-Glutamine (L-Glu), and 1% penicillin/streptomycin and maintained in 5% CO_2_ at 37°C.

HEK293T cells were cultured in DMEM supplemented with 10% Tet-system approved FBS, 1% penicillin/streptomycin, and 5% L-Glutamine (L-Glu). The doxycycline-inducible cells were generated by lentiviral infection.

### Method details

#### Preparation of egg extracts

*Xenopus* interphase egg extracts and sperm nuclei were prepared as described here and in previous work.^10^ *Xenopus* eggs were collected in 100 mM NaCl from chorionic gonadotropin-injected female frogs. The eggs were incubated in de-jelling buffer (10 mM Tris pH 8.5, 110 mM NaCl, 5 mM DTT), rinsed three times in MMR (100 mM HEPES-KOH, pH 7.5, 2 M NaCl, 10 mM KCl, 5 mM MgSO_4_, 10 mM CaCl_2_, 0.5 mM EDTA) and activated with 5 μM Calcium Ionophore (Sigma-Aldrich, Cat. No. C7522). The activated eggs were washed with MMR and then rinsed three times with an ice-cold S-buffer (50 mM HEPES-KOH pH 7.5, 50 mM KCl, 2.5 mM MgCl_2_, 250 mM sucrose) supplemented with 2 mM β-mercaptoethanol and 15 μg/mL leupeptin (Sigma-Aldrich, Cat. No. L2023). Eggs were packed by spinning to remove the excess buffer and then crushed at 13,000 rpm for 12 min at 4°C. The crude extract was collected, supplemented with cytochalasin B (40 μg/mL) (Sigma-Aldrich, Cat. No. C6762), and centrifuged at 70,000 rpm for 18 min at 4°C in a TLA100.3 rotor (Beckman Optima).

#### DNA templates

Demembranated sperm nuclei were prepared as described here and in previous work.^10^ *X. laevis* males were primed with 50U Folligon 7 days in advance and with 300U HCG the day before the sperm dissection. Dissected testis were collected in EB buffer (50 mM HEPES-KOH, pH 7.6, 50 mM KCl, 5 mM MgCl_2_, 5 mM EGTA, 2 mM β-mercaptoethanol). Testis were rinsed three times in ice-cold EB buffer and finely chopped with a razor blade. The material was then transferred to a 15 mL Falcon tube and spun for 5 min in a swinging-bucket rotor (JS13.1, Beckman) at 4,250 × g at 4°C. The pellet was then resuspended in 1.5 mL of SuNaSp buffer at room temperature [0.25 M sucrose (Sigma-Aldrich, Cat. No. S0389), 75 mM NaCl, 0.5 mM spermidine (Sigma-Aldrich, Cat. No. S0266), 0.15 mM spermine (Sigma-Aldrich, Cat. No. S4264)]. To remove membranes lysolecithin 2 mg/mL (Sigma-Aldrich, Cat. No. L4129) was added and incubated for 10 min at room temperature. The reaction was stopped by adding 3% BSA (Sigma-Aldrich, Cat. No. A7030). The pellet was resuspended again in 1 mL ice-cold EB and spun at 2,000 × g for 5 min at 4°C. The final pellet was resuspended in 400 μL of EB in 30% glycerol.

#### Chromatin isolation from egg extract

To isolate chromatin fractions, sperm DNA (3000 nuclei/μL) was added to 30 μL egg extracts and incubated at 23°C for the indicated time. Samples were diluted with 10 volumes of ice-cold EB (100 mM KCl, 2.5 mM MgCl_2,_ and 50 mM HEPES-KOH pH 7.5) containing 0.25% NP-40 and centrifuged through a 0.5 M sucrose layer at 10.000 × g for 5 min at 4°C. Pellets were washed once with EB and suspended in SDS-PAGE sample buffer. Proteins were then resolved by SDS-PAGE and monitored by WB.

#### Replication reactions

DNA replication in *Xenopus* egg extract was performed as described here and in previous work.^46^ Sperm nuclei (3000 nuclei/μL) were added to 20 μL S-phase egg extract supplemented with 0.1 μL of α-^32^P-dCTP (250 mCi; 3000 Ci/mmol) and incubated at 23°C. At the indicated time points, reactions were stopped with Stop buffer (8 mM EDTA, 80 mM Tris pH 8.0, 1% w/v SDS), supplemented with 1 mg/mL Proteinase K, and incubated at 50°C for 2 h. Samples were treated with RNase (0.6 mg/mL) to degrade any trace of RNA and purified DNA was then separated from the unincorporated label by electrophoresis through a 0.8% agarose gel in TBE 1×. The gel was fixed in 30% TCA for 20 min, dried, and exposed for autoradiography.

For quantification of DNA replication, the gel was exposed to a phosphoscreen (Cytiva). The recorded radioactive signals were monitored within a phosphoImager (Typhoon) and measured with ImageQuant software.

#### Preparation of recombinant proteins

For *in vitro* studies, full-length *Xenopus laevis* CDC6 (rCDC6) was cloned in pGEX-6P-1 vector and GST-rXCDC6 was expressed in Rosetta (DE3) competent cells (Novagen) by growing transformed cells to OD_600_ 0.7–0.8 at 37°C, then lowering the temperature to 18°C, and inducing protein expression overnight with 0.1 mM IPTG addition. Cells were subsequently harvested by centrifugation at 5.000 × *g*, 15 min, at 4°C, resuspended in ice-cold lysis buffer (PBS 1×, 100 mM KCl, 1 mM MgCl_2_, 1 mM DTT, 10% glycerol, 40 mL per liter of cell culture) supplemented with protease inhibitors Cocktail Set III (Calbiochem, Cat. No. 539134), and lysed by sonication. Lysates were centrifuged at 40.000 × *g*, 60 min, at 4°C, to eliminate cell debris, and cleared lysates were incubated for 1 h at 4°C with 4 mL slurry Glutathione Sepharose 4 Fast Flow resin (Cytiva) per liter of cell culture. Beads were harvested, and flow through was incubated a second time with other 4 mL of glutathione beads, for 1 h at 4°C. Beads from the two incubations were pulled and washed with lysis buffer, then HRV 3C protease was added to enable cleavage of GST from rCDC6 for calorimetric experiments. The reaction was carried out overnight at 4°C. Supernatant, containing cleaved rCDC6, was diluted 3 times with buffer A (10 mM NaH_2_PO_4_ K_2_HPO_4_, 2.7 mM KCl, 5% Glycerol, 1 mM DTT) and loaded on a Resource S 6 mL column (Cytiva) equilibrated in buffer A, using high salt buffer B (PBS 1×, NaCl 860 mM, 2.7 mM KCl, 5% Glycerol, 1 mM DTT) for protein elution. A 20-column volume, 8–30% gradient was applied; fractions containing rCDC6, as judged by SDS-PAGE analysis, were pooled, concentrated using a Vivaspin device (Sartorius), and loaded on a Superdex 200 10/300 column (Cytiva) equilibrated in SEC buffer (PBS 1×, 10% glycerol, 1 mM MgCl_2_, 1 mM DTT). Peak fractions from size exclusion chromatography were pooled, concentrated, and stored at −80°C after snap freezing in liquid nitrogen. Protein concentration was determined by absorbance at 280 nm using calculated extinction. The *Xenopus* CDC6 vector (Glutathion-S-Transferase (GST)-tagged *Xenopus* Cdc6) was a gift from M. Méchali^47^ (The Institute of Human Genetics, University of Montpellier).

#### Isothermal titration calorimetry

Experiments were done at 25°C using a PEAQ-ITC MicroCalorimeter (MicroCal, Malvern Instruments Ltd., Malvern, UK) following the general procedure.^19^ A 75 μM rCDC6 solution was titrated with NSC-95397 compound (1 mM) using injections of 2 μL. Protein and ligand were prepared and diluted to be in the same final buffer (SEC buffer with 1% DMSO). Calorimetric data were analyzed with the instrument software. Heat change peaks were integrated and normalized per mole of NSC-95397 injected. The ITC binding curve was fitted to the one binding site model equation.

#### *Xenopus laevis* embryos and microinjections

*X. laevis* embryos were obtained by *in vitro* fertilization of freshly laid *X. laevis* eggs with crushed *X. laevis* testes. Only batches with greater than 90% fertilization efficiency were used. Twenty minutes after fertilization, embryos were de-jellied in 2% cysteine (Sigma-Aldrich, Cat. No. 30089) pH 8.1 dissolved in 0.1xMBS. Microinjections were performed using calibrated needles and embryos equilibrated in 1xMBS/3% Nicoll PM-400 (Sigma-Aldrich, Cat. No. F4375). Microinjection needles were generated from borosilicate glass capillaries (Harvard Apparatus, GC 100F-15) using the micropipette puller Sutter p97. Maximally 4 nL of mRNA were injected into de-jellied embryos at the 1-cell stage using the microinjector PicoSpritzer III (Parker). After the first cleavage, the buffer was replaced with 1xMBS/2% Ficoll then 0.1xMBS, and embryos were allowed to develop to the desired stages. Embryos were staged according to Newport and Faber, 1975.

For *in vitro* transcription, DNA encoding Full-length Flag-SSRP1 1-709aa^9^ and *X. laevis* CDC6 (XlCDC6) were cloned between the FseI and AscI sites of pCS2 vector (pCS2-6xMYC_SSRP1, pCS2-6xMYC_xCDC6). To obtain sense RNA from these constructs, plasmids were linearized with NotI and KpnI, respectively. Following linearization, mRNA was expressed from the SP6 promoter using the mMessage mMachine kit (ThermoFisher Scientific, Cat. No. AM1340). All constructs were verified by DNA sequencing.

The pCS2 vector was a gift from Philip Zegerman (The Gurdon Institute, University of Cambridge).

#### Lentiviral vectors

The lentiviral doxycycline-inducible pCW57.1-6xMYC_SSRP1 plasmid was generated using Gateway Gene Cloning System (ThermoFisher Scientific), according to the manufacturer’s instructions. 6xMYC_SSRP1 insert was PCR amplified from pCS2-6xMYC_SSRP1 using the following primers:

attb1_6M_for:

5′-GGGGACAAGTTTGTACAAAAAAGCAGGCTACGCGTGCAGGATCCCATCGATTTAAAGCTAC

CATG-3’;

attb1_6M_rev:

5′-GGGGACCACTTTGTACAAGAAAGCTGGGTACCGGTCCTCCCACACCTCCCCCTG-3’.

The PCR reaction was then used directly in the BP recombination reaction with the pDONR221 vector. Correct pDONR plasmid was used for the LR recombination reaction with pCW57.1 as the destination vector (Addgene, #41393). All constructs were verified by DNA sequencing.

#### Imaging of embryos

Embryo development was documented upon drug treatment using a Nikon SMZ1500 stereomicroscope coupled to a Nikon Ds-Fi1-U2 color camera at ×2 magnification with fiber optic illumination using NIS-Elements software. Embryos were imaged by placing them in a dish filled with water.

#### Protein analysis

Embryos lysates were prepared as described here and in previous work.^9^ Embryos were lysed in 50 μL/embryo lysis buffer (50 mM Tris pH 7.6, 150 mM NaCl, 10 mM EDTA, 1% Triton X-100) supplemented with Protease Inhibitor Cocktail Set III (Calbiochem, Cat. No. 539134), incubated on ice for 30 min, sonicated for 15 min on High setting 30s ON/OFF using Bioruptor Next Gen (Diagenode) in a water bath at 4°C, and centrifuged at max speed for 30 min at 4°C.

To prepare total cell extracts, cell pellets were washed twice in PBS and then lysed in RIPA buffer (50 mM Tris-HCl pH 7.5, 150 mM NaCl, 0.1% SDS, 0.5% NaDeoxycholate, 1% NP-40, 5 mM EDTA pH 8.0) supplemented with Protease Inhibitor Cocktail Set III, incubated on ice for 30 min, sonicated for 15 min on High setting 30s ON/OFF using Bioruptor Next Gen (Diagenode) in a water bath at 4°C, and centrifuged at max speed for 30 min at 4°C. Protein concentration was determined using Biorad protein assay according to the manufacturer’s instructions (Bio-Rad Laboratories).

#### Antibodies and western blot analysis

Samples were resolved by 4–20% SDS-PAGE and analyzed by standard WB techniques. Blots were probed using the following antibodies: rabbit polyclonal anti-*Xenopus* Cdc45 antibody, mouse monoclonal anti-*Xenopus* Orc1 antibody, mouse monoclonal anti-Cdk1 (A17), and mouse monoclonal anti-Cdk1 pTyr15 were obtained from J. Gannon (The Francis Crick Institute, London, UK) and rabbit polyclonal anti-*Xenopus* H2A.X-F1, obtained by D. Shechter, A. Einstein Institute, New York, NY, USA. Mouse monoclonal anti-MCM7 (Santa Cruz Biotechnology, sc-9966), anti-Cdt1 (F-6, Santa Cruz Biotechnology, sc-365305), rabbit polyclonal anti-Psf3,^43^ mouse monoclonal anti-Ssrp1 (10D7, Abcam, ab26212), rabbit polyclonal anti-Cdc6 (Santa Cruz Biotechnology, sc-8341), mouse monoclonal anti-Polα (p180), obtained from Abmart (clone 13026-1-3/C199), mouse monoclonal anti-Polδ, obtained from Abmart (clone 19570-1-1/C316),^44^ rabbit polyclonal anti-H2B (Millipore, 07–371), anti-H3 (Abcam, ab1791), mouse monoclonal anti-Rad51 (Abcam, ab213), and HRP anti-Myc tag antibody (9E10) (Abcam, ab62928). Detection with secondary antibodies was commonly carried out at 1:5000. Proteins were detected by WesternBright ECL (Advansta) on Bio-Rad ChemiDoc XRS+ or by iBright FL1500 Imaging system (ThermoFisher Scientific).

#### Materials *Xenopus* extract preparation for ELISA assay

Cycloheximide (Calbiochem, Cat. No. 239763) was dissolved in water at 10 mg/mL, creatine phosphate (Roche, Cat. No. 10621714001) was dissolved in water at 1 M. Creatine phosphokinase (Sigma-Aldrich, Cat. No. C3755) was dissolved in water at 10 mg/mL. RL5a (Sigma-Aldrich, Cat. No. SML2187) was dissolved in 100% DMSO at 20 mM. Recombinant geminin use was previously described.^42^

#### Small-molecule library

The compound library used for the screening contained 3828 small molecules. In detail, the library was assembled starting from collections of different vendors: FDA-approved and drug-like molecules were from Selleck Chemicals, Sigma-Aldrich, and MicroSource; kinase-targeted molecules were from Asinex, Enamine, BioFocus, ChemBridge, and Chem-X; targeting protein-protein interaction molecules were from Asinex. The screening run set was prepared by combining small molecules in groups of four, for a total of 957 multiplexed chemical entities. Equal volumes of 10 mM DMSO preparations were mixed (2.5 mM final concentration for each compound) and stored at 4°C in Nunc 384-well polypropylene plates (ThermoFisher Scientific, Cat. No. 4312).

#### High throughput assay and compound screen

A biotinylated DNA (bDNA, 1.8 kb) was generated using a nested PCR approach to conduct the ELISA-based screening. Briefly, the plasmid pBlueScript KS(−) was used as a template in the first round of amplification to generate a larger amplicon (2205 bp) using the following primers:

pBS_NEST_For: 5′- GTGCACGAGTGGGTTACATC -3’;

pBS_NEST_Rev: 5′- CTCTTCGCTATTACGCCAGC -3'.

This molecule served as a template in a subsequent series of amplifications to produce the bDNA used for the screening using the following primers:

pBS_b_For1: 5’-[BtnTg]CACAACATACGAGCCGGAAG-3’;

pBS_Rev1: 5′-GTGCACGAGTGGGTTACATC-3'.

PCR products were pooled and subjected to a purification and concentration step with the Wizard SV Gel and PCR Clean-Up System (Promega, Cat. No. A9281). bDNA was eluted in TE buffer (10 mM Tris-HCl pH 7.5, and 1 mM EDTA) and stored at −20°C.

For plate coating, bDNA was diluted to 20 ng/μL in TEN buffer (TE buffer, 1M NaCl), dispensed (50 μL/well) in Pierce 384-well Streptavidin-coated plates (ThermoFisher Scientific, Cat. No. 15505) and incubated for 2 h at room temperature. Wells were washed twice with TE buffer and filled with 50 μL of ice-cold TE. Plates were then sealed and stored at 4°C for a maximum of one week. On the day of the screening, 384-well bDNA coated plates were washed with modified ice-cold EB (mEB) buffer (10 mM HEPES, pH 7.5, 2.5 mM MgCl_2_, 50 mM KCl) and kept on ice until use.

To exclude any variability that could derive from different *X. laevis* preparations, snap-frozen aliquots of extracts were quickly thawed, pooled, and centrifuged at 3,000 × g for 1 h at 4°C. The supernatant was diluted 1:2 with ice-cold mEB buffer, supplemented with cycloheximide (100 μg/mL), creatine phosphate (30 mM), and creatine phosphokinase (150 μg/mL), and collected in a 50 mL Falcon tube.

For drug treatment, compounds were assayed at a higher concentration (187 μM) than in conventional biochemical or cellular screening due to the very high concentration of lipids and proteins (50 μg/μL) in *X. laevis* extracts. 40 μL of extracts were automatically dispensed into bDNA-coated 384-well plates using a Hamilton Microlab STAR Liquid Handling robot. 3 μL of multiplexed library compounds were added, mixed thoroughly, and incubated at 23°C on a thermomixer for 30 min. After incubation, plates were washed twice with ice-cold mEB buffer, and the DNA: protein complexes were crosslinked by incubating for 5 min on ice with mEB buffer containing 0.5% formaldehyde (50 μL). The amount of MCM-7 bound to biotinylated DNA was measured by ELISA. Solutions and washes were removed by manual aspiration, followed by gently tapping the plate, inverted, onto a paper towel. Briefly, plates were washed twice with TBS and blocked with 5% BSA-TBS-Tween20 0.05% (TBST) for 1 h at RT. The blocking solution was replaced with 50 μL of 5% BSA-TBST containing anti-MCM-7 antibody at dilution of 1:100 (Santa Cruz, Cat. No. sc-9966). Following incubation (2 h) and washes with TBST, HRP-labeled secondary antibody (1:3000) was incubated for 1 h at RT. The plates were then washed three times with TBST. 50 μL of a chemiluminescent substrate (SuperSignal ELISA Pico, ThermoFisher Scientific, Cat. No. 37069) were added to each well and the signal was acquired on an EnSpire plate reader (PerkinElmer).

Analysis of the raw data of primary screening (z-scores determination) was performed using the HiTSeekR *online* platform.^45^

#### Fluorescent intercalator displacement assay

To reach the final tested concentration, 3 μL of compounds were added to a 57 μL mixture of bDNA (100 ng) and SYBR green reagent dissolved in 10 mM Tris-HCl, 1 mM EDTA pH 8,0 for a total volume reaction of 60 μL. Fluorescence readings were taken on an Enspire Spectrophotometer (PerkinElmer). Five scans per sample were taken every 5 min for 20 min with an excitation wavelength of 485 nM and an emission wavelength of 528 nM. The level of fluorescence change (either enhancement or quenching) was determined and converted to a percent change in fluorescence using the formula (F1/F0) ∗100, where F0 is the initial fluorescence of dye bound to DNA before the addition of drug and F1 is the fluorescence after addition of drug.

#### NSC-95397 analogs

Analogs of NSC-95397 were bought from different vendors. In details, IFM_47059 (Cat. No. 016562) and IFM_47060 (Cat. No. 016561) were bought from Matrix Scientific, IFM_47061 (Cat. No. STK249723) and IFM_47064 (Cat. No. STK235850) were bought from VitasMlab, IFM_47062 was bought from Sigma Aldrich (Cat. No. SML0367), IFM_47063 was bought from AMS Private Supplier (Cat. No. A001210844) and IFM_47065 was bought from AChemBlock (Cat. No. O32425).

#### Chemical inhibition

Embryos at the two-cell stage were divided into a dish containing 5 mL control or treatment solutions and then incubated at 22°C and kept in the dark to prevent chemical degradation related to light exposure. The NSC-95397 was used at a concentration of 200 μM. Control embryos were treated with 0.1% DMSO. Work solutions of NSC-95397 were prepared by diluting the stock solution prepared in DMSO in 0.1 x MMR (All work solutions contained 0.1% DMSO).

#### Preparation of lentiviral supernatants and transduction of cells

Lentiviral particles containing pCW57.1-6xMYC_SSRP1 or the pCW57.1-Empty vector (EV) were generated in HEK293T cells using Lipofectamine 2000 Transfection Reagent (ThermoFisher Scientific). HEK293T cells were grown on 10-cm plates to 70–80% confluence and co-transfected with 10 μg of each lentiviral plasmid [pCW57.1 (Empty vector), pCW57.1_6xMYC_SSRP1], 4 μg VSV-G plasmid DNA and 10 μg packaging viral CMV-Δ8.9 plasmid. The viral supernatant was harvested at 48 h, and 72 h post-transfection, filtered through a 0.45 μm filter, and immediately used to infect recipient cells. A few days after infection, cells were selected with puromycin for 5 days.

#### Crystal Violet assay

Each cell line was seeded in 48-well plates at a density of 25 × 10^3^ cells/well, and SSRP1 overexpression was induced with 1 μg/mL Doxycycline. After 24 h, cells were treated with increasing concentrations of NSC-95397 (0.25, 0.5, 1, 2.5, and 5 μM). DMSO solvent without compound (0 μM) served as a negative control. The plates were incubated for an additional 48 h at 37°C.

For IC50 experiments, each cell line was seeded in 96-well plates at a density of 2 × 10^3^ cells/well, and SSRP1 overexpression was induced with 1 μg/mL Doxycycline. After 24 h, cells were treated with increasing concentrations of NSC-95397 (0.01, 0.025, 0.05, 0.1, 0.25, 0.5, 1, 2.5, 10 and 25 μM). DMSO solvent without compound (0 μM) served as a negative control. The plates were incubated for an additional 72 h at 37°C. On the day of staining, the culture medium was removed and the cells were rinsed once with PBS and stained with 1% Crystal Violet solution for 20 min. Excess dye was discarded by washing with water and cells were dried on filter paper. The cell-bound dye was dissolved in 10% glacial acetic acid for 30 min while shaking gently on a rocking shaker and the absorbance of the solubilized CV was measured at 570 nm with a VICTOR3 V 1420 Multilabel plate reader (PerkinElmer Wallac). All proliferation assays were performed in biological triplicates normalized to the untreated control (100%) and error bars represent the mean ± standard error of the mean (SEM).

#### Immunofluorescence microscopy

Sperm nuclei (3000/μL) incubated in *X. laevis* interphase egg extract were supplemented with Cy3-dCTP (Amersham Biosciences, Cat. No. PA53021) for the indicated times at 23°C with DMSO, used as a control, or 100-140-180-200 μM NSC-95397. Samples were fixed and mounted between a slide and a coverslip in fixing solution (45 mM PIPES at pH 7.2, 45 mM NaCl, 240 mM KCl, 10% formalin, 50% glycerol, 2 μg mL^−1^ Hoechst and 3,3-dihexyloxacarbocyanine (DioC6)). Fluorescence images were captured with fluorescence microscopy. Areas of Hoechst-stained nuclei were used to define the region of interest (ROI) using ImageJ. The Cy3-dCTP staining intensity of each ROI was measured and shown as the Integrated Density of selected nuclei.

### Quantification and statistical analysis

All experiments, if not indicated otherwise in the figure legend, were performed three times and representative experiments are depicted. No statistical methods or criteria were used to estimate sample size or to include/exclude samples. Statistical analysis was performed with GraphPad PRISM software (version 9.3.0) and Microsoft Excel. The analysis of variance (ANOVA) tests were used to compare each of several groups’ means against a single control group and assess pairwise comparisons between group means. ∗∗∗∗ = *p* < 0.0001, ∗∗∗ = *p* < 0.001, ∗∗ = *p* < 0.01 and ns = non significant indicate significance. Image analysis was conducted using ImageJ. Statistical details for each experiment including sample size, significance *p* values, and test performed are indicated in figure legends and figures.
