# Supplementary material for: A high-throughput screening identifies MCM chromatin loading inhibitors targeting cells with increased replication origins
Source: iScience. 2024 Jul 22;27(8):110567. doi: 10.1016/j.isci.2024.110567 (PMC11342271; doi:10.1016/j.isci.2024.110567)
Supplement: Document S1. Figures S1–S5 [file mmc1.pdf]

iScience, Volume *27*

Supplemental information

A high-throughput screening identifies MCM chromatin loading inhibitors targeting cells with increased replication origins

Lucia Falbo, Hervé Técher, Vincenzo Sannino, Michela Robusto, Giovanni Fagà, Federica Pezzimenti, Francesco Romeo, Luca Gabriele Colombo, Stefania Vultaggio, Daniele Fancelli, Silvia Monzani, Valentina Cecatiello, Sebastiano Pasqualato, Mario Varasi, Ciro Mercurio, and Vincenzo Costanzo

Figure S1

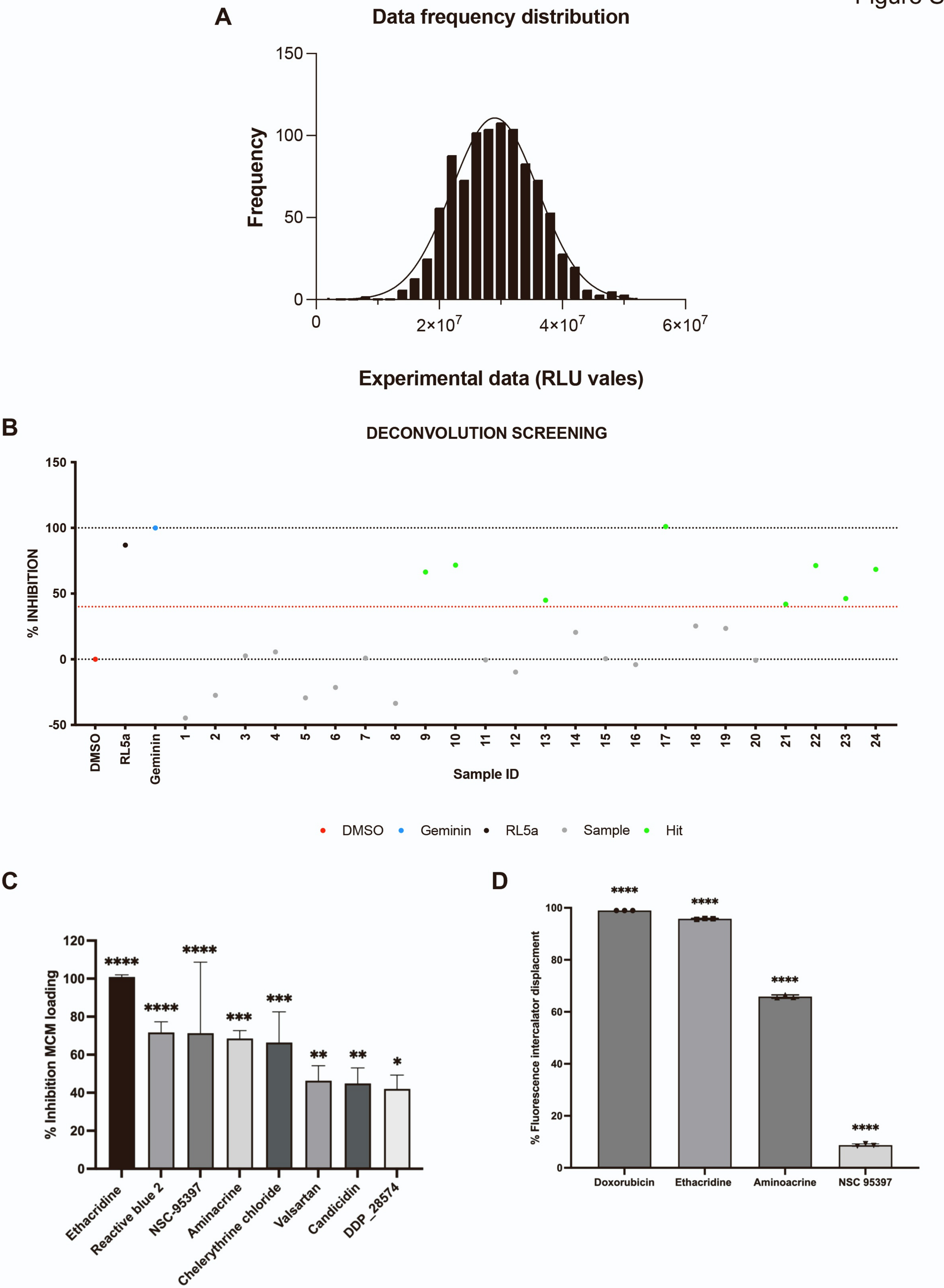
A
Data frequency distribution
Frequency
150
100
50
0
0
2×10^7
4×10^7
6×10^7
Experimental data (RLU vales)
B
DECONVOLUTION SCREENING
% INHIBITION
150
100
50
0
-50
DMSO
RL5a
Geminin
1
2
3
4
5
6
7
8
9
10
11
12
13
14
15
16
17
18
19
20
21
22
23
24
Sample ID
DMSO
Geminin
RL5a
Sample
Hit
C
% Inhibition MCM loading
120
100
80
60
40
20
0
****
****
****
***
***
**
**
*
Ethacridine
Reactive blue 2
NSC-95397
Aminacrine
Chelerythrine chloride
Valsartan
Candicidin
DDP_28574
D
% Fluorescence intercalator displacment
100
80
60
40
20
0
****
****
****
****
Doxorubicin
Ethacridine
Aminoacrine
NSC 95397

Figure S1. Data frequency distribution and deconvolution, related to Figures 2 and 3.
A. Data frequency distribution of the primary screening. The graph illustrates the standard normal distribution curve of the luminescence data of the samples of the primary screening. Data are reported as Relative Luminescence Unit (RLU).
B. Deconvolution screening. The 24 compounds that composed the 6 pools that emerged as hits in the confirmation screening were tested in triplicate. Data are expressed as the mean ± standard deviation (SD) of the percentage of inhibition of MCM7 loading on bDNA compared to vehicle (DMSO).
C. Screening of single compounds on the inhibition of MCM loading expressed as percentage relative to DMSO performed in triplicate. Data are expressed as the mean ± standard deviation (SD). One-way analysis of variance (ANOVA) test was used to analyze differences among multiple groups compared to the control (DMSO). *=$P < 0.0169$, **=$P < 0.0078$ (Valsartan), **=$P < 0.0100$ (Candicin), ***=$P = 0.0001$ (Aminacrine), ***=$P = 0.0002$ (Chelerythrine chloride), ****=$P < 0.0001$.
D. Fluorescence intercalator displacement (FID) assay. Ethacridine, Aminoacrine, and NSC-95397 were analyzed for the ability to displace a fluorescent SYBR green DNA intercalator as a measure of the direct interaction of the compound with DNA. Doxorubicin was used as a positive control and tested at a final concentration of 2 µM. Ethacridine and Aminoacrine were tested at 100 µM, while NSC-95397 was tested at a final concentration of 200 µM. Data were analyzed using GraphPad Prism 9.0 and shown as mean ± standard deviation (SD). One-way analysis of variance (ANOVA) test was used to analyze differences among multiple groups compared to the control (DMSO). ****=$P < 0.0001$.

Figure S2

A

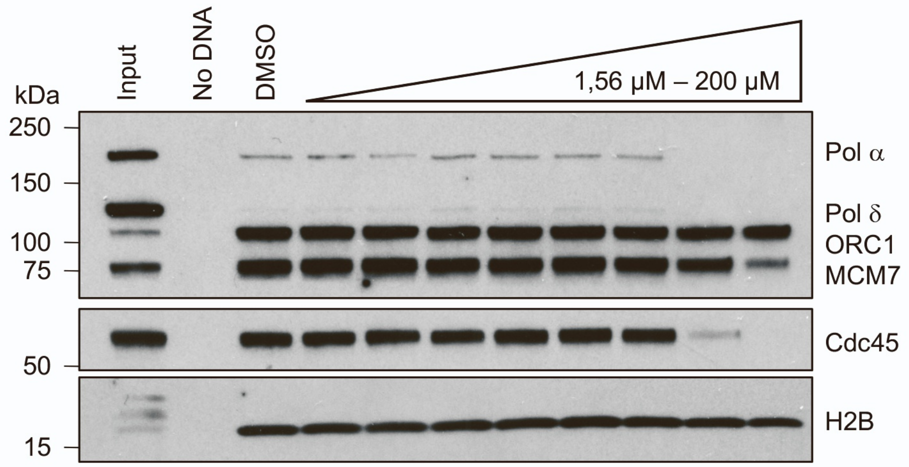

Input
No DNA
DMSO
1,56 µM – 200 µM
kDa
250
150
100
75
50
15
Pol α
Pol δ
ORC1
MCM7
Cdc45
H2B

B

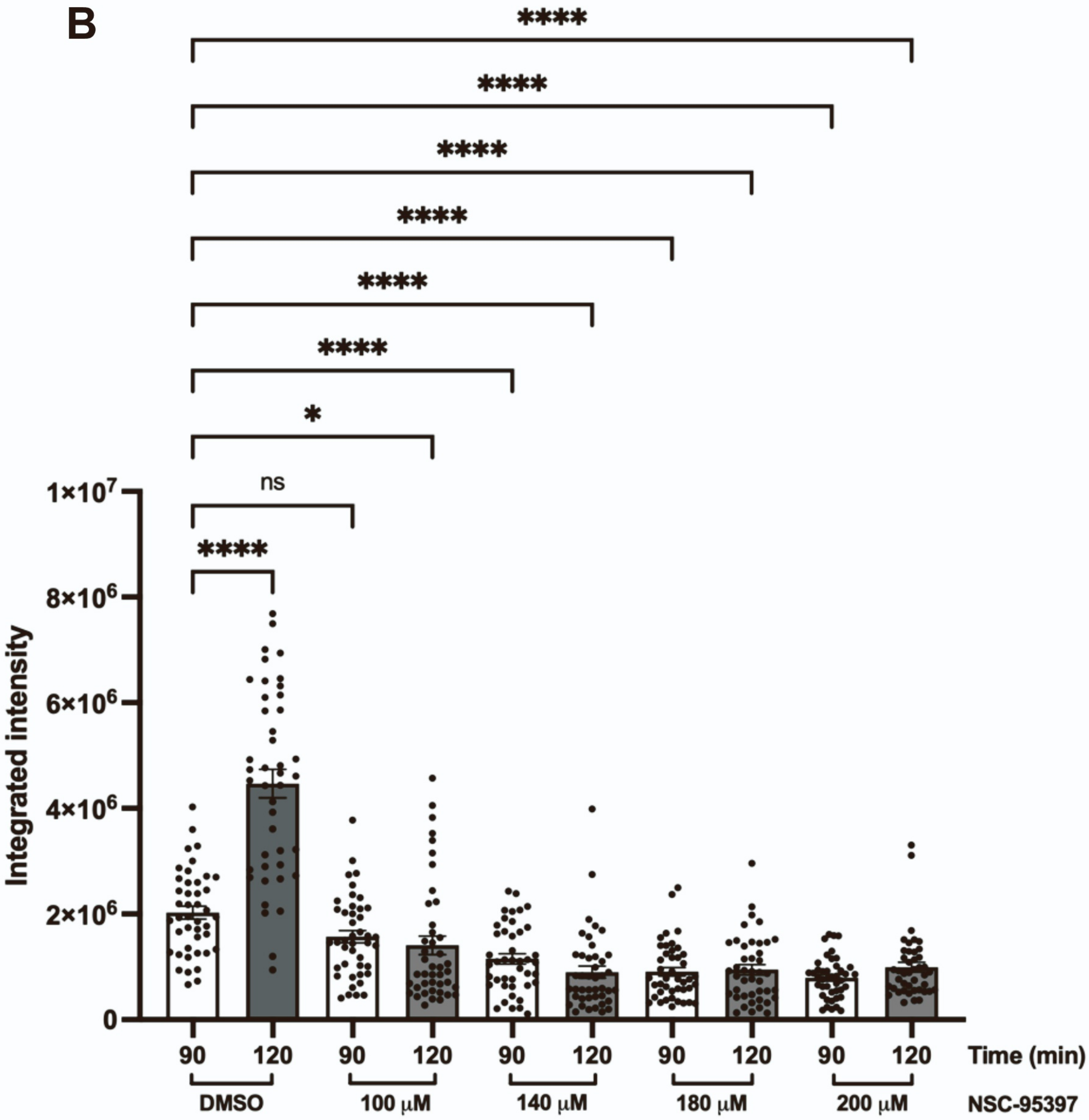

****
****
****
****
****
****
*
ns
****
Integrated intensity
1×10^7
8×10^6
6×10^6
4×10^6
2×10^6
0
90
120
90
120
90
120
90
120
90
120
Time (min)
DMSO
100 µM
140 µM
180 µM
200 µM
NSC-95397

Figure S2. NSC-95397 effects on chromatin and nuclei, related to Figure 3.
A. Western Blot analysis of the indicated proteins bound to chromatin isolated from sperm nuclei incubated with increasing concentration of NSC-95397 or DMSO, used as Control.
B. The Cy3-dCTP incorporation of more than 40 nuclei was quantified as fluorescence-integrated intensity per nucleus using ImageJ. Data are represented as scatter dot plots with mean ± SEM. A One-way ANOVA test was used to perform multiple comparisons compared to DMSO at 90 min, used as a control. *=P = 0.0112, ****=P < 0.0001, ns = not significant.

Figure S3

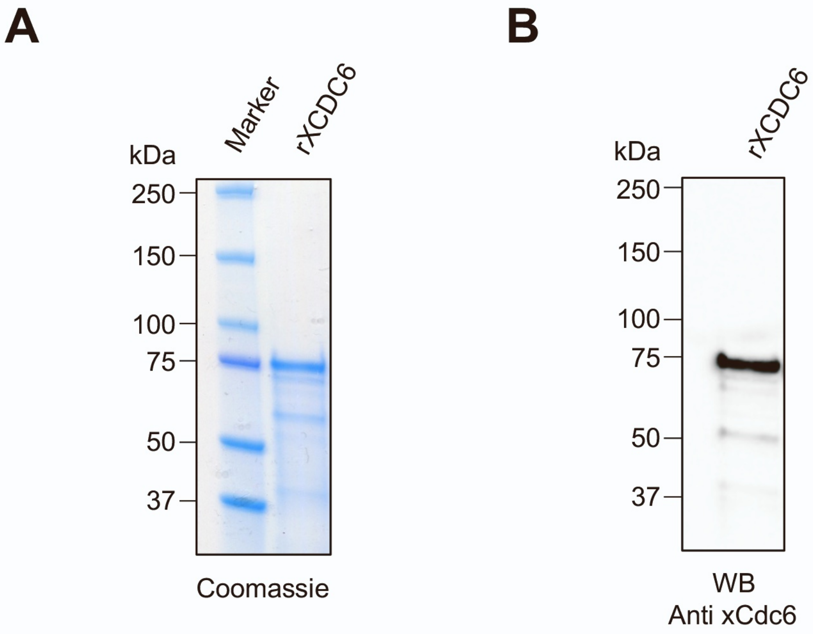
A
Marker
rXCDC6
kDa
250
150
100
75
50
37
Coomassie
B
rXCDC6
kDa
250
150
100
75
50
37
WB
Anti xCdc6

Figure S3. Recombinant *Xenopus* CDC6, related to Figure 4.
A. Coomassie blue-stained SDS/PAGE gel of purified recombinant CDC6 protein.
B. Western Blot of purified recombinant *Xenopus* CDC6 protein.

Figure S4

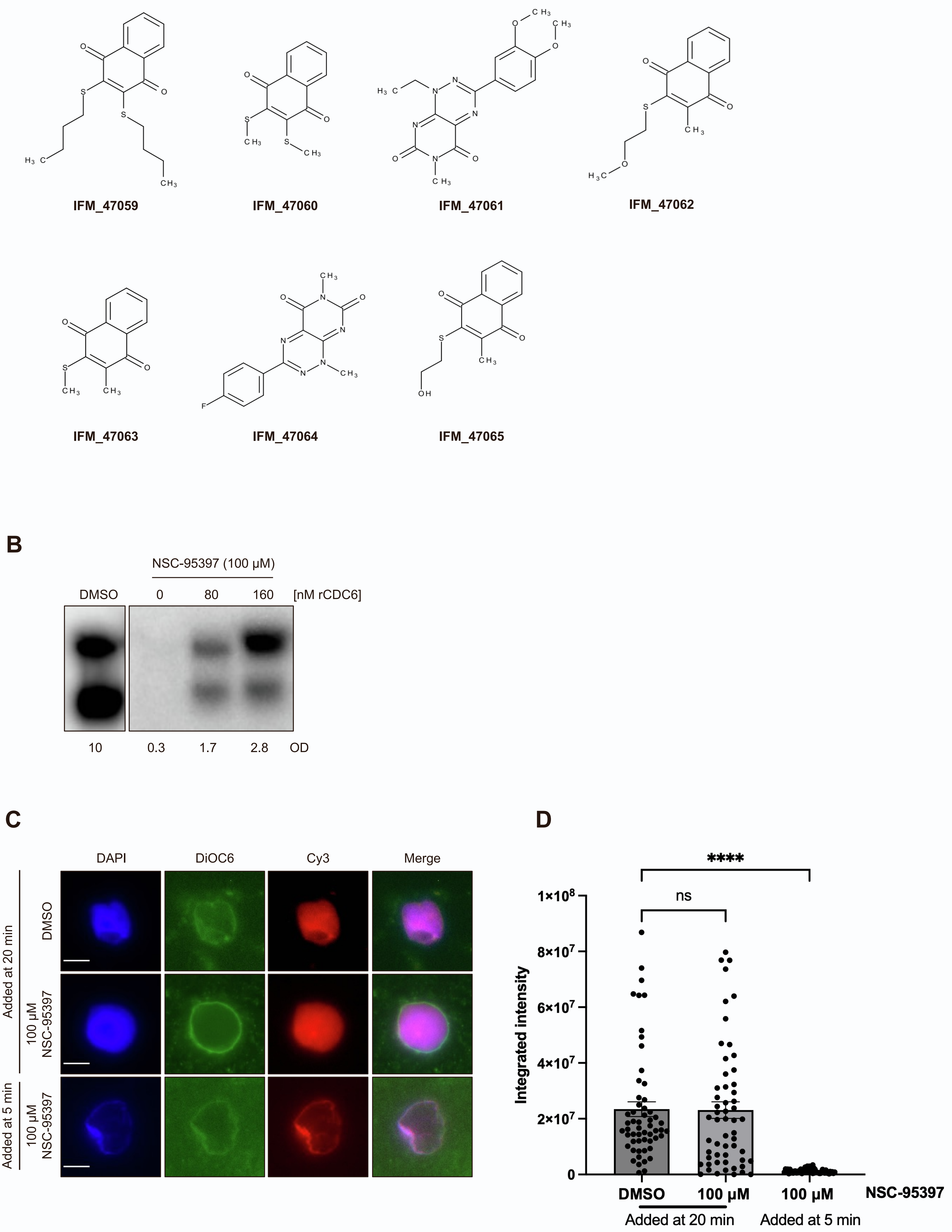
A
IFM_47059
IFM_47060
IFM_47061
IFM_47062
IFM_47063
IFM_47064
IFM_47065
B
NSC-95397 (100 μM)
DMSO
0
80
160
[nM rCDC6]
10
0.3
1.7
2.8
OD
C
DAPI
DiOC6
Cy3
Merge
Added at 20 min
DMSO
100 μM NSC-95397
Added at 5 min
100 μM NSC-95397
D
****
ns
Integrated intensity
1×10^8
8×10^7
6×10^7
4×10^7
2×10^7
0
DMSO
100 μM
100 μM
NSC-95397
Added at 20 min
Added at 5 min

Figure S4. Rescue with recombinant *Xenopus* CDC6, related to Figure 4.

A. Chemical structure of the NSC-95397 analogs (IFM_47059, IFM_47060, IFM_47061, IFM_47062, IFM_47063, IFM_47064, IFM_47065).

B. Representative autoradiography of a DNA replication assay showing α^{32}P-dCTP incorporation for 90 min in sperm nuclei. Recombinant *Xenopus laevis* CDC6 was added to egg extract treated with DMSO or 100 µM NSC-95397 at a level similar to that of the endogenous protein (80 nM) and in double amount (160 nM). Optical density (OD) for each lane is indicated. Samples derived from the same gel.

C. Representative images of sperm nuclei incubated in interphase extract supplemented with Cy3-dCTP (red) for 30 min and treated with DMSO, used as control, or 100 µM NSC-95397 at 5 or 20 min from nuclei addition to egg extract. Samples were fixed and stained with 4,6-diamidino-2-phenylindole (DAPI) for DNA (blue) and DiOC6 (green) for membranes. Scale bar = 10 µm.

D. The Cy3-dCTP incorporation of 55 nuclei was quantified as fluorescence-integrated intensity per nucleus using ImageJ. Data are represented as scatter dot plots with mean ± SEM. A two-way ANOVA test was used to perform multiple comparisons relative to DMSO, used as a negative control. ****=$P < 0.0001$, ns = not significant.

Figure S5

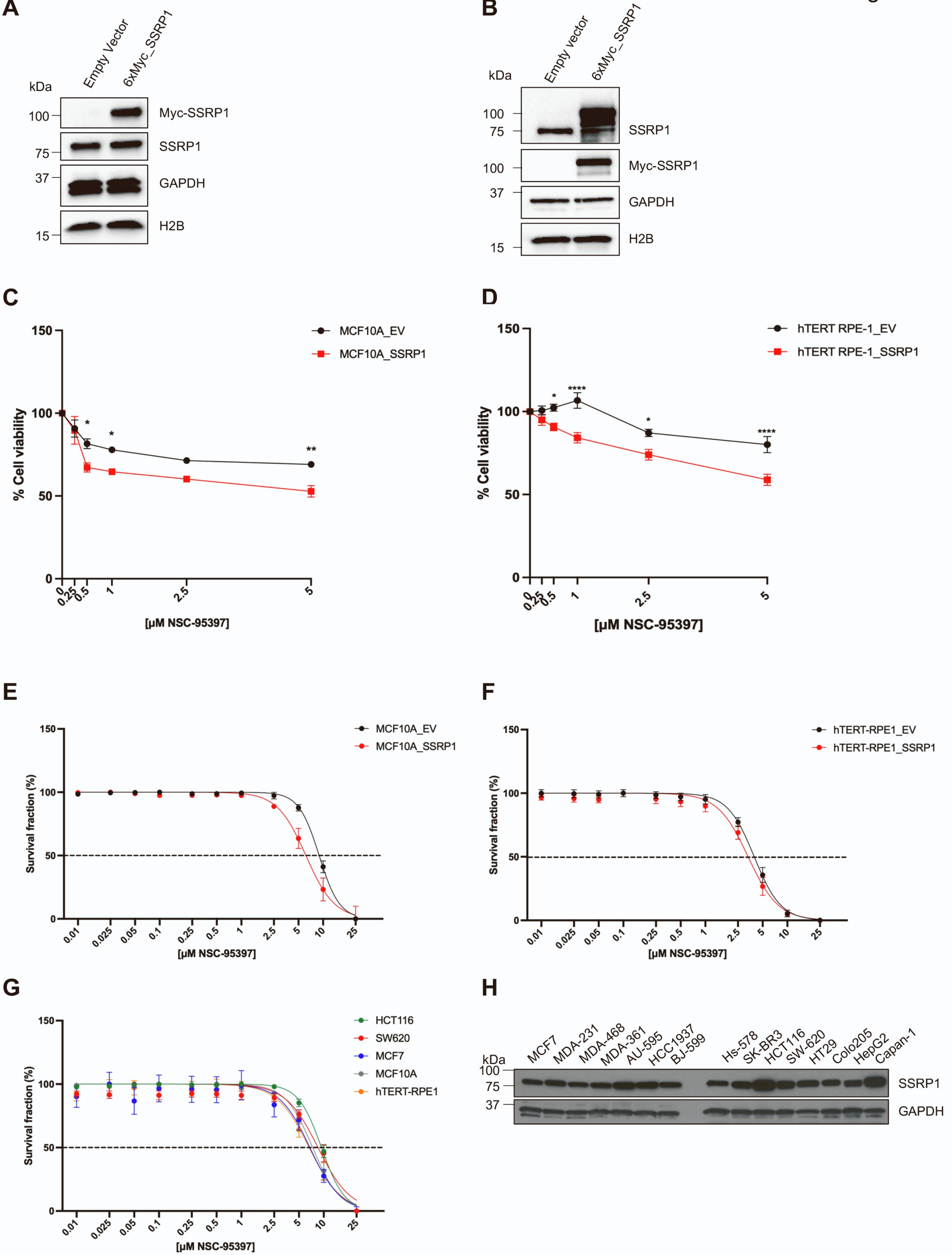
A
Empty Vector
6xMyc_SSRP1
kDa
100
75
37
15
Myc-SSRP1
SSRP1
GAPDH
H2B
B
Empty vector
6xMyc_SSRP1
kDa
100
75
100
37
15
SSRP1
Myc-SSRP1
GAPDH
H2B
C
MCF10A_EV
MCF10A_SSRP1
% Cell viability
[µM NSC-95397]
D
hTERT RPE-1_EV
hTERT RPE-1_SSRP1
% Cell viability
[µM NSC-95397]
E
MCF10A_EV
MCF10A_SSRP1
Survival fraction (%)
[µM NSC-95397]
F
hTERT-RPE1_EV
hTERT-RPE1_SSRP1
Survival fraction (%)
[µM NSC-95397]
G
HCT116
SW620
MCF7
MCF10A
hTERT-RPE1
Survival fraction (%)
[µM NSC-95397]
H
MCF7
MDA-231
MDA-468
MDA-361
AU-595
HCC1937
BJ-599
Hs-578
SK-BR3
HCT116
SW-620
HT29
Colo205
HepG2
Capan-1
kDa
100
75
37
SSRP1
GAPDH

Figure S5. Effects of NSC-95397 compound on *in vitro* cell proliferation, related to Figure 6.
A. Western Blot showing Doxycycline (Dox)-induced expression of Myc-SSRP1 in MCF10A cells. GAPDH was used as a loading control.
B. Western Blot showing Doxycycline (Dox)-induced expression of Myc-SSRP1 in hTERT RPE-1 cells. GAPDH was used as a loading control.
C. Dose-response curves of human MCF10A cells treated with increasing concentrations of NSC-95397 for 48 hrs determined by the Crystal Violet staining assay. Cytotoxicity is reported as the percentage of cell viability relative to DMSO-treated cells (0 μM). A two-way ANOVA test was used to perform multiple comparisons relative to the control (DMSO). Data are represented as mean ± SEM. *=P = 0.0195 (0.5 μM), *=P = 0.0354 (1 μM) ,**=$P < 0.0046$.
D. Dose-response curves of human hTERT RPE-1 cells treated with increasing concentrations of NSC-95397 for 48 hrs determined by the Crystal Violet staining assay. Cytotoxicity is reported as the percentage of cell viability relative to DMSO-treated cells (0 μM). A two-way ANOVA test was used to perform multiple comparisons relative to the control (DMSO). Data are represented as mean ± SEM. *=P = 0.0449 (0.5 μM), *=P = 0.0149 (2.5 μM),****=$P < 0.0001$.
E. Dose-response curves of human MCF10A clones treated with increasing concentrations of NSC-95397 for 72 hrs determined by the Crystal Violet staining assay. Cytotoxicity is reported as the percentage of cell viability relative to DMSO-treated cells (0 μM). Data represents two independent experiments and are shown as mean and error ± SEM.
F. Dose-response curves of human hTERT-RPE1 clones treated with increasing concentrations of NSC-95397 for 72 hrs determined by the Crystal Violet staining assay. Cytotoxicity is reported as the percentage of cell viability relative to DMSO-treated cells (0 μM). Data represents three independent experiments and are shown as mean and error ± SEM.
G. Dose-response curves of human MCF7 (blue), MCF10A (grey), HCT116 (green), SW620 (red), and hTERT-RPE1 (orange) cells treated with increasing concentrations of NSC-95397 for 72 hrs determined by the Crystal Violet staining assay. Cytotoxicity is reported as the percentage of survival fraction relative to DMSO-treated cells (0 μM). Data represents six independent experiments and are shown as mean and error ± SEM. Colors indicate cell type.
H. Western blot showing endogenous expression of SSRP1 in MCF7, HCT116, SW620 cells. GAPDH was used as a loading control.
